# Supplementary material for: Reversible thermal regulation for bifunctional dynamic control of gene expression in Escherichia coli
Source: Nat Commun. 2021 Mar 3;12:1411. doi: 10.1038/s41467-021-21654-x (PMC7930084; doi:10.1038/s41467-021-21654-x)
Supplement: Supplementary file 1 — Supplementary Information [file 41467_2021_21654_MOESM1_ESM.pdf]

Supplementary Info for

Reversible thermal regulation for bifunctional dynamic control of gene expression in *Escherichia coli*

Authors:

Xuan Wang[1,2,#], Jia-Ning Han[1,#], Xu Zhang[1], Yue-Yuan Ma[1], Yina Lin[1], Huan Wang[1], Dian-Jie Li[5], Tao-Ran Zheng[1], Fu-Qing Wu[1,3], Jian-Wen Ye[1,3,4*], Guo-Qiang Chen[1,2,3*]

Affiliations:

[1] Center for Synthetic and Systems Biology, School of Life Sciences, Tsinghua University, Beijing 100084, China

[2] Tsinghua-Peking Center for Life Sciences, Beijing 100084, China

[3] MOE Key Lab of Industrial Biocatalysts, Department of Chemical Engineering, Tsinghua University, Beijing 100084, China

[4] Center for Materials Synthetic Biology, CAS Key Laboratory of Quantitative Engineering Biology, Shenzhen Institute of Synthetic Biology, Shenzhen Institute of Advanced Technology, Chinese Academy of Sciences, Shenzhen, 518055, China

[5] School of Physics, Peking University, Beijing 100871, China

[#] These authors contributed equally in this study

Co-corresponding authors*:

Jian-Wen Ye: jw.ye@siat.ac.cn (Ye JW)

Shenzhen Institute of Synthetic Biology, Shenzhen Institute of Advanced Technology, Chinese Academy of Sciences, Shenzhen, 518055, China

Guo-Qiang Chen: chengq@mail.tsinghua.edu.cn (Chen GQ);

School of Life Sciences, Tsinghua University, Beijing 100084, China.

Phone: +86-10-62783844; Fax: +86-10-62794217

Supplementary Info:

Supplementary Methods....................4

Supplementary Table 1 Strains and genes used in this study....................6

Supplementary Table 2 Primiers and gene sequence for *lacI* deletion and degradation tag sequences used in this study....................7

Supplementary Table 3 Thermodynamic and mechanical characterization of various PHA materials....................8

Supplementary Figure 1 Gene *lacI* deletion in *E. coli* JM109SG....................9

Supplementary Figure 2 Comparative analysis of T-switch performance in LB and M9 medium. 10

Supplementary Figure 3 Design and characterization of T-switch derivates carrying degradation tags. 11

Supplementary Figure 4 mRNA levels in *E. coli* and its recombinant harboring T-switch circuits at 30℃ and 37℃, respectively....................12

Supplementary Figure 5 RNA-seq profiling. 13

Supplementary Figure 6 Switch-testing of ON- and OFF-responses in different growth phases. 14

Supplementary Figure 7 Fluorescent distribution of constructs 147+168 in different growth phases 15

Supplementary Figure 8 Correlations between different designs of the *phlF-mrfp* expression module....................16

Supplementary Figure 9 Online monitoring of cells containing T-switch constructs from 37℃ to 30℃....................17

Supplementary Figure 10 Tree-ring like colony formation by *E. coli* harboring T-switch, constructs 147+167. 18

Supplementary Figure 11 Tree-ring like colony formation by *E. coli* harboring T-switch, constructs 147+168. 19

Supplementary Figure 12 Growth effects on tree ring-like colony formation....................22

Supplementary Figure 13 Schematics of homopolymer, random- and block-copolymers composed of 3HB and 4HB monomers....................23

Supplementary Figure 14 Cell growth studies in different culture scales....................24

Supplementary Figure 15 Expression debugging of 3HB and 4HB synthsis pathways. 25

Supplementary Figure 16 Microbial synthesis of block copolymers PHB-*b*-P4HB in a fermentor. 27

Supplementary Figure 17 NMR study on D-values of PHB-*b*-P4HB produced from fed-batch fermentations. 28

Supplementary Figure 18 DSC thermodynamic characterization of PHB-*b*-P4HB. 29

Supplementary Figure 19 NMR study on D-values of PHB-*b*-P4HB produced from fed-batch fermentations. 30

Supplementary Figure 20 Microbial synthesis of random copolymer P(3HB-*co*-4HB) by recombinant *E. coli* grown in a fermentor. 31

Supplementary Figure 21 NMR study on D-values of P(3HB-*co*-4HB) from fed-batch fermentations. 33

Supplementary Figure 22 DSC thermodynamic assays of P(3HB-*co*-4HB). 34

Supplementary Methods

Gene *lacI* knockout

Recombinant *E. coli* deleted with *sad* and *gabD* genes encoding two types of semi-aldehyde dehydrogenase, was constructed to enhance 4HB synthesis flux via weakening bypasses, resulting in *E. coli* JM109SG. Here, gene *lacI* encoding repressor LacI to repress synthetic *tac* promoter P_{tac}, was deleted from the genome of *E. coli* JM109SG to form *E. coli* JM109SGL, which serves as a chassis in this study. Approach of PCR-mediated gene deletion was used to delete gene *lacI* in *E. coli* JM109SG. Gene deletion was confirmed by PCR analysis and DNA sequencing.

RNA-seq profiling

To test the genome-wide effects of T-switch, the primary design composed of constructs 155+165 encoded in recombinant *E. coli* were grown at 30□ (TS_30) and 37□ (TS_37) in 500 mL shake flasks containing 50 mL LB medium supplemented with 20 g L^{-1} glucose, respectively, for RNA-seq analysis. The start chassis *E. coli* JM109SGL cultured at 30 and 37□, respectively, were employed as controls. Total RNA of these four groups were harvested from their respective cultures grown in their mid-log phase at an OD_{600} between 2.9-3.1. Cultures were centrifuged at 3,000 g at 4□ for 10 min, cell precipitates washed with ice-bath ddH_2O twice. Precipitated cells were stored at a -80□ freezer prior to the RNA extraction. Finally, samples were sent to Bionova (Beijing, China) for total RNA extraction, RNA-seq analysis and then normalized reads of genes (RPKM, Reads Per Kilobase per Million mapped reads). Logorithmic values of expression fold changes in control groups (37 vs. 30) and experimental groups (TS_37 vs. TS_30), namely $\log_2(FC_{37/30})$ and $\log_2(FC_{TS\_37/TS\_30})$, were used to study the temperature effects on gene expression. The ratio of $\log_2(FC_{TS\_37/TS\_30})$ to $\log_2(FC_{37/30})$ was employed to study the effects of T-switch expression probably resulted from CI^{857} and PhlF repressors (Supplementary Figures. 5b-c). Illumina HiSeq 2500 was used for DNA sequencing here.

Cell growth characterization during various growth phases

To characterize the cell growth of recombinant *E. coli* carrying T-switch constructs in different cultural scales, on- and/or off-line measurements of OD_{600} (optical density at 600 nm) were carried out in a 200 μL 96 microplate containing 100 μL LB medium, 250 mL shake flasks containing 20 mL LB medium and 7 L bench-top bioreactor containing 3 L LB medium, respectively. *E. coli* JM109SGL and its recombinant harboring T-switch (155+165), were inoculated into 1 mL LB medium using single colonies as inoculums for a 12 h growth (1, 000

rpm, Thermal Shaker, AOSHENG, China) at 30□ and 37□, as seed cultures, respectively. Subsequently, seed cultures were diluted with a fresh LB medium to an OD_{600} of 0.05 in 300 µL 96 microplate wells and 250 mL shake flasks, respectively, for a 16 h growth analysis. Procedures of seed preparation and cell cultivation in 1 L bioreactor were the same as described for PHA fed-batch fermentation (See Methods). Specifically, OD_{600} was measured every 30 min by a microplate reader (Varioskan Flash，Thermal Fisher, USA) at 1,000 rpm. In contrast, OD_{600} of cell cultures in 250 mL shake flasks and 7 L bioreactor were measured every 2 h via off-line sampling.

Mechanical properties assays

PHA films were cut to form dumbbell-shape specimens using a cutting machine (RR/PCP, Ray-Ran, England) with a width of 3 mm and a thickness of approximately 60-180 µm. The stress-strain measurements of samples were analyzed using a tensile machine (Instron 3365, INSTRON, USA) at room temperature. The stretch speed was 10-50 mm min^{-1}. Elongation at break (nominal strain at break) (%)[1] was calculated by the distance between grips, of which is 10 mm. For accuracy, each experimental group contained three parallel samples.

M9 medium

In order to characterize the T-switch (construct 155+165) performance in chemical defined culture medium, fluorescent intensity of harvested cells grown in minimal medium (M9) in the same condition was compared with that collected from LB medium. The M9 medium is consisted of 1mM thiamine hydrochloride, 0.4% glucose, 0.2% BD casamino acids, 2mM $MgSO_4$, and 0.1mM $CaCl_2$ in 1 L 5X M9 salts solution, which contains 64 g Na_2HPO_4•$7H_2O$, 15 g KH_2PO_4, 2.5 g NaCl and 5 g NH_4Cl dissolved in 1 L deionized water.

Supplementary Table 1 Strains and genes used in this study

Strains/ Genes	Description	References
**strains**		
E. coli JM109SG	Start chassis derived from *E. coli* JM109 with deficiency of succinate semialdehyde dehydrogenases encoded by *gabD* and *sad* gene, respectively	2
JM109SGL	Derivates of JM109SG by deleting *lacI* gene	In this study
**Genes**		
phaC	PHA synthase of *Rostonia eutropha* H16 (*R. eutropha*)	2
phaB	Acetoacetyl-CoA reductase of *R. eutropha*	2
phaA	β-Ketothiolase of *R. eutropha*	2
4hbd	4-Hydroxybutyrate dehydrogenase of *Clostridium kluyveri* (*C. kluyveri*)	3
sucD	Succinate semialdehyde dehydrogenase of *C. kluyveri*	3
orfZ	4-Hydroxybutyrate-CoA transferase of *C. kluyveri*	3
gabD	Endogenous succinate semialdehyde dehydrogenase encoded by *gabD* in *E. coli* JM109	3
sad	Endogenous succinate semialdehyde dehydrogenase encoded by *sad* in *E. coli* JM109	3
mreB	Endougenous cell shape-determining actin co-ordinates cell wall morphologenesis, sphere shape of recombinant cells can be achieved by overexpressing or deleting *mreB*	4
ftsZ	Encoding cell division protein, FtsZ, allowing the formation of fibre-shape recombinant cells	4
lacI	IPTG-responsive regulator controlling the transcription of P_{tac} promoter	5
cI857	Thermo-sensitive regulator forming monomer and dimer reversibly under different temperatures from 28 to 42 □	6
phlF	A *tetR* family repressor enabling a tight repression of promoter P_{PhlF}	7
mrfp	Red fluorescent protein, mcherry RFP, as a reporter	8
sfgfp	Super-fold green fluorescent protein, sfGFP, as a reporter	9
LVA	Degradation tag listed in Supplementary Table 2	10
AAV	Degradation tag listed in Supplementary Table 2	10

Supplementary Table 2 Primiers and gene sequence for *lacI* deletion and degradation tag sequences used in this study

Gene & Primers & degradation tag	Nucleotide Sequence
lacI	GACACCATCGAATGGCGCAAAACCTTTCGCGGTATGGCATG<br>ATAGCGCCCGGAAGAGAGTCAATTCAGGGTGGTGAATGTGA<br>AACCAGTAACGTTATACGATGTCGCAGAGTATGCCGGTGTCT<br>CTTATCAGACCGTTTCCCGCGTGGTGAACCAGGCCAGCCACG<br>TTTCTGCGAAAACGCGGGAAAAAGTGGAAGCGGCGATGGCG<br>GAGCTGAATTACATTCCCAACCGCGTGGCACAACAACTGGC<br>GGGCAAACAGTCGTTGCTGATTGGCGTTGCCACCTCCAGTCT<br>GGCCCTGCACGCGCCGTCGCAAATTGTCGCGGCGATTAAAT<br>CTCGCGCCGATCAACTGGGTGCCAGCGTGGTGGTGTCGATG<br>GTAGAACGAAGCGGCGTCGAAGCCTGTAAAGCGGCGGTGCA<br>CAATCTTCTCGCGCAACGCGTCAGTGGGCTGATCATTAACTA<br>TCCGCTGGATGACCAGGATGCCATTGCTGTGGAAGCTGCCTG<br>CACTAATGTTCCGGCGTTATTTCTTGATGTCTCTGACCAGAC<br>ACCCATCAACAGTATTATTTTCTCCCATGAAGACGGTACGCG<br>ACTGGGCGTGGAGCATCTGGTCGCATTGGGTCACCAGCAAA<br>TCGCGCTGTTAGCGGGCCCATTAAGTTCTGTCTCGGCGCGTC<br>TGCGTCTGGCTGGCTGGCATAAATATCTCACTCGCAATCAAA<br>TTCAGCCGATAGCGGAACGGGAAGGCGACTGGAGTGCCATG<br>TCCGGTTTTCAACAAACCATGCAAATGCTGAATGAGGGCAT<br>CGTTCCCACTGCGATGCTGGTTGCCAACGATCAGATGGCGCT<br>GGGCGCAATGCGCGCCATTACCGAGTCCGGGCTGCGCGTTG<br>GTGCGGATATCTCGGTAGTGGGATACGACGATACCGAAGAC<br>AGCTCATGTTATATCCCGCCGTTAACCACCATCAAACAGGAT<br>TTTCGCCTGCTGGGGCAAACCAGCGTGGACCGCTTGCTGCAA<br>CTCTCTCAGGGCCAGGCGGTGAAGGGCAATCAGCTGTTGCC<br>CGTCTCACTGGTGAAAAGAAAAACCACCCTGGCGCCCAATA<br>CGCAAACCGCCTCTCCCCGCGCGTTGGCCGATTCATTAATGC<br>AGCTGGCACGACAGGTTTCCCGACTGGAAAGCGGGCAGTGA
Primers-in for PCR varification	In-VF: TCACTGCCCGCTTTCCAGTCGG<br>In-VR: GACACCATCGAATGGCGCAAAACC
Primers-out or PCR varification	Out-VF: CCACACAACATACGAGCCGGAAGCA<br>Out-VR: CTGGTGTATATGGCGAGCGCAATGAC
LVA	ACTAGTGCAGCAAACGACGAAAACTACGCTTTAGTAGCT<br>(**TSAANDENYALVA**)
AAV	CCTGCTGCAAACGACGAAAACTACGCTGCAGCAGTT<br>(**PAANDENYAAAV**)

Letters in blue are promoter sequences, red (GTG) are start codons of *lacI*. **bold** are amino acids sequences of protein degradation tags used in this study.

Supplementary Table 3 Thermodynamic and mechanical characterization of various PHA materials

	T_g (□)	T_m (□)	Young's modulus (MPa) [a]	Tensile stress (MPa) [a]	Elongation at break (%)[d]
PHB	n.d.	170.66	222.2 ± 14.5	6.4 ± 0.6	5.7 ± 0.6
P(3HB-*co*-30% 4HB)	-19.0	n.d.[b]	3.6 ± 0.3	4.8 ± 0.2	983.63 ± 31.54
P(3HB-*co*-64% 4HB)	-40.2	n.d.[b]	1.73 ± 0.3	8.9 ± 0.2	1180.60 ± 70.26
P(3HB-*co*-89% 4HB)	-43.0	41.9	28.4 ± 3.7	41.2 ± 1.3	1087.34 ± 24.01
PHB-*b*-69.7% P4HB	-44.8	49.3/167.3	40.2 ± 2.3	24.8 ± 0.4	852.34 ± 5.89
P4HB	-44.9	51.3	/ [c]	/ [c]	

For thermodynamic characterization, data were obtained from non-isothermal melt crystallization and subsequent heating scans. Samples weighted 5~8 mg were sealed in aluminum pans, heated to 190□ at a rate of 40□ min^{-1} and held at 190□ for 2 min to eliminate previous thermal histories. Subsequently, they were cooled to −50□ at a rate of 10□ min^{-1} and reheated to 190□ at a rate of 10□ min^{-1}. For mechanical assays, PHA films were cut into dumbbell-shape specimens for stress-strain measurements.

[a] The thickness of PHA films for measuring Young's modulus and Tensile strength was adjusted to approximately 70-170 μm.

[b] Melting temperature (T_m) were not observed during the data processing.

[c] Because of a lack of P4HB homopolymer, its mechanical property assays were not available.

[d] Elongation at break (nominal strain at break)(%)[1] was calculated from the distance between grips.

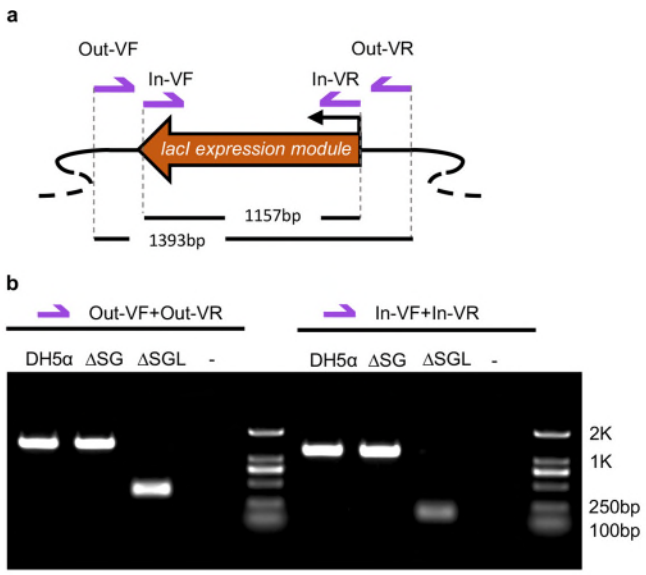

Supplementary Figure 1 Gene *lacI* deletion in *E. coli* JM109SG.

(**a**) Primer design for deleting *lacI* expression module including promoter and coding sequence regions, 1157 base pairs in total. Primer sequences are listed in Supplementary Table 2. (**b**) Double-check of *lacI* gene deletion by PCR varification using two groups of primers, Out-VF/R and in-VF/R. *E. coli* DH5α, an *E. coli* k12 derived strain, and *E. coli* JM109SG, were used as negative controls, respectively. '-' indicates blank groups. PCR verification was done for three times independently.

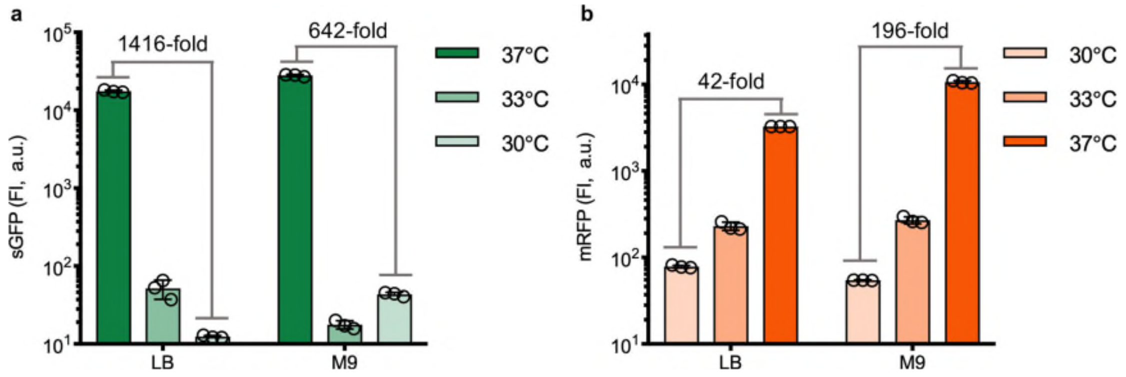

Supplementary Figure 2 Comparative analysis of T-switch performance in LB and M9 medium.

The recombiant cells harboring T-switch (construct 155+165) grown in the LB and M9 media, respectively, at 30□, 33□ and 37°C, were harvested for fluorescence measurement of sfGFP (**a**) or mRFP (**b**) by FACS (see methods). Error bars, mean ± s.d. of three replicates (data points in circle). FI, Fluorescence Intensity in arbitrary unit (a.u.).

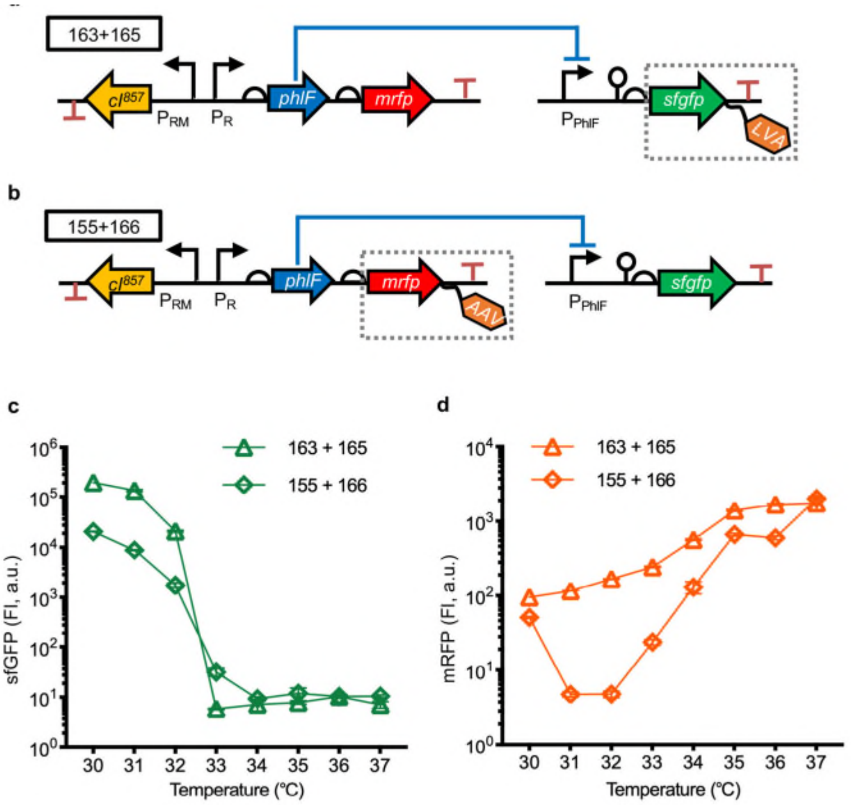

Supplementary Figure 3 Design and characterization of T-switch derivates carrying degradation tags.

(**a**) and (**b**) are constructs of T-switches derived from 155+165 group by adding degradation tag LVA to *sfgfp* and AAV to *mrfp*, respectively, forming 163+165 and 155+166 groups . (**c**) and (**d**) displayed temperature-responsive function of T-switch derivates, constructs 163+165 and 155+166, via cytometer analysis. Compared to the T-switch of 155+165 group shown in Figure 1, the fluorescence intensity of mRFP maintained considerable performance in on-stage at 37℃ and exihibited significant decrease in off-stage at low temperatures except at 30℃. For sfGFP expression, the low temperature-responded on-stage had a higher fluorescence intensity by linking LVA tag and showed sharply decrease from 32℃ to 33℃ with a tight control of off-stage from 33℃ to 37℃.

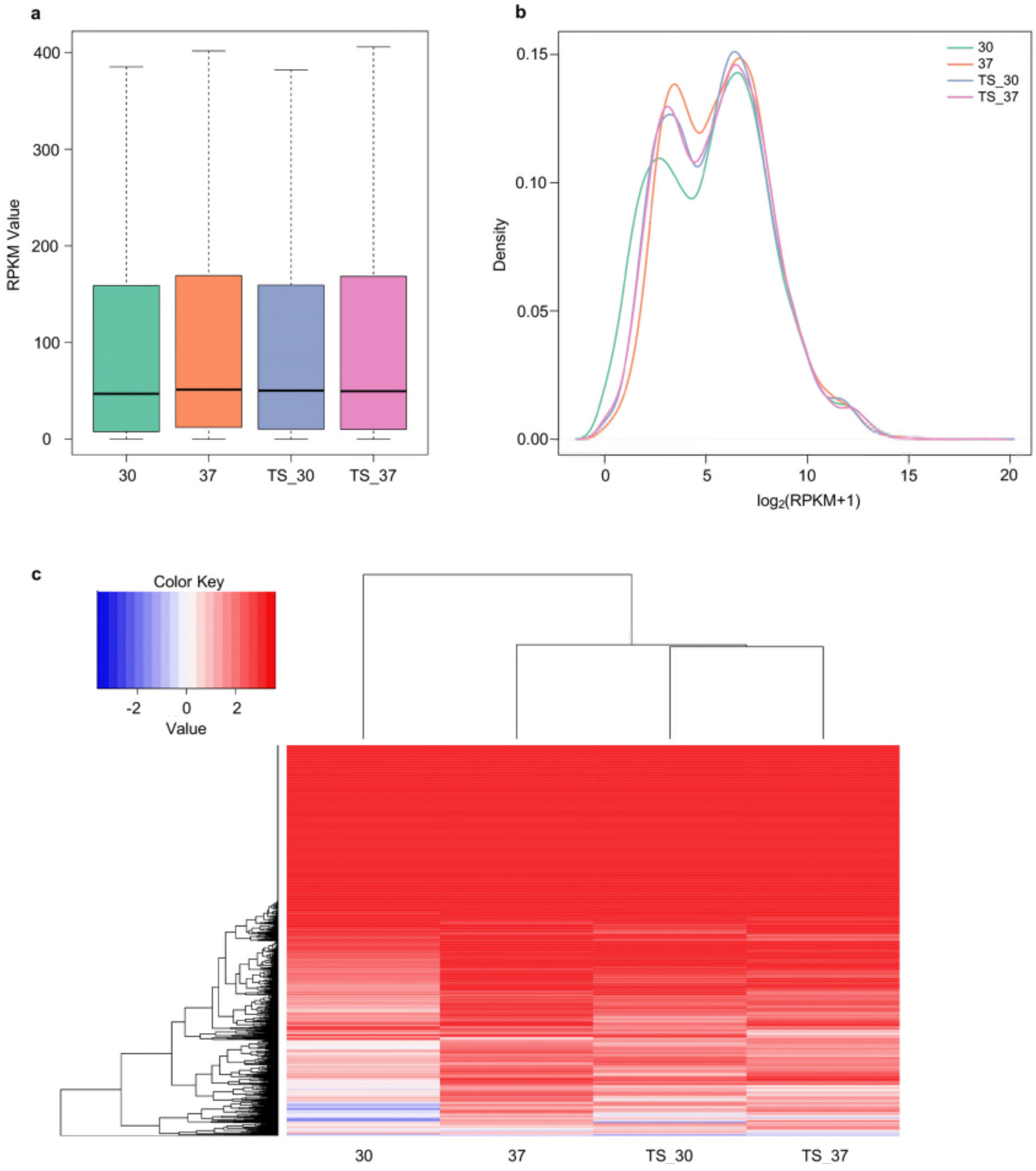

Supplementary Figure 4 mRNA levels in *E. coli* and its recombinant harboring T-switch circuits at 30□ and 37□, respectively.

(**a**) Reads Per Kilobase Millon Mapped Reads (RPKM, see Supplementary methods) distribution of four samples, including *E. coli* JM109SGL cultured at 30□ and 37□, namely 30 and 37 groups, and *E. coli* JM109SGL harboring T-switch circuits cultured at 30□ and 37□, namely TS_30 and TS_37 groups shown in *x*-axis, the value of RPKM is plotted in *y*-axis. (**b**) Density distribution of genome-wide gene expression. The *y*-axis represents density, and *x*-axis is the value of $\log_2$(RPKM+1). (**c**) Heatmap of mRNA levels clustering cross different samples using Hierarchical Cluster. The change of color from blue to red indicates the expression levels from low to high.

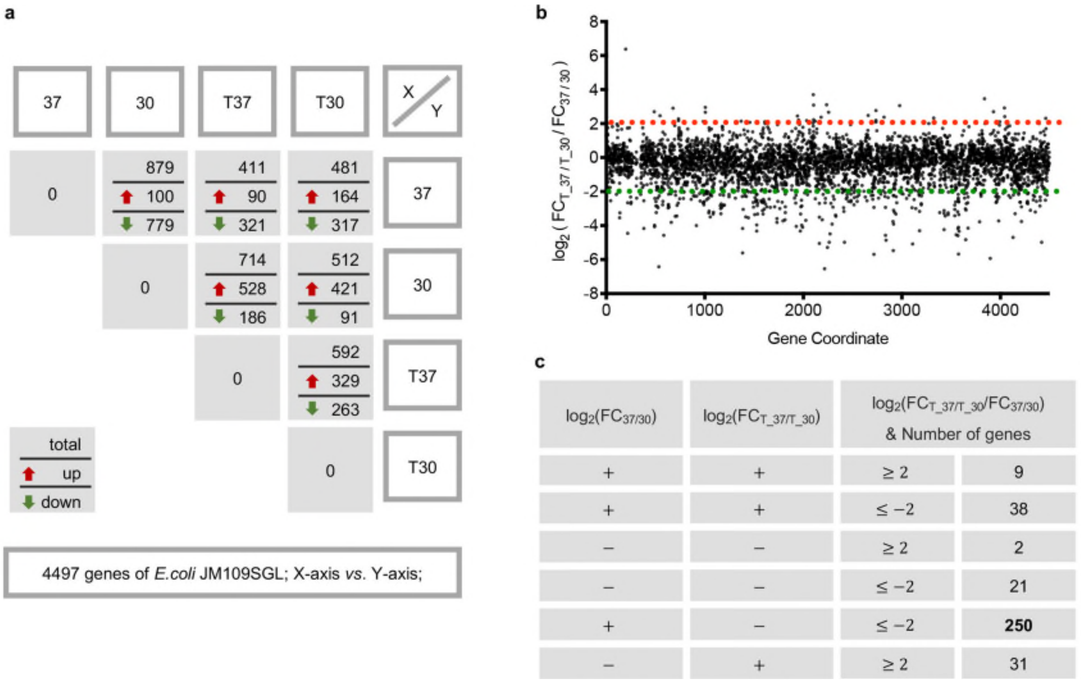

Supplementary Figure 5 RNA-seq profiling.

(**a**) A comparison of mRNA levels of *E. coli* JM109SGL and its recombinant harboring T-switch constructs, 155+165, cultured in shake flasks at 30□ and 37□, respectively. Genes of differential abundant, of which the value of |fold change| (FC) is greater than two between the two comparative groups, with a RPKM over 0.1 a.u. and q-value lower than 0.01, in either strain, are summarized including numbers of up-regulated (red arrow) and down-regulated (green arrow) genes, as well as the sum of them. Detailed information was listed in Source data file. (**b**) Displayed the |fold change| distribution against gene coordinate, and (**c**) showed comparative analysis results with a smaller size of gene set with significant variance in mRNA levels, having the value of |fold change| greater than four between two comparative groups, as well as a RPKM higher than 0.1 a.u. and q-value lower than 0.01, in either strain by removing the noises resulted from temperature between 30□ and 37□ (see Supplementary Methods).

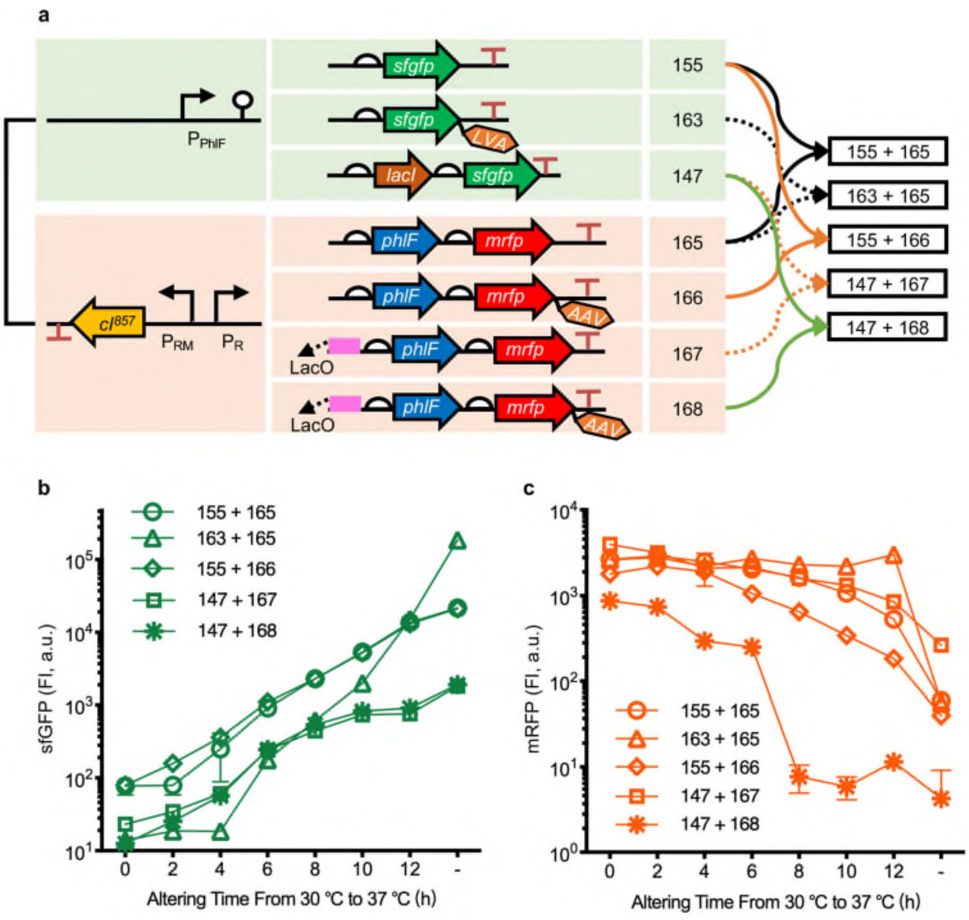

Supplementary Figure 6 Switch-testing of ON- and OFF-responses in different growth phases.

(**a**) Construct of T-switch, 155+165 group, and its derivates by introducing negative feedback loop control (LacO) of input signal (*lacI-sfgfp*), and adding degradation tags to reporters of sfGFP and mRFP, respectively. Specifically, *lacI* gene was co-expressed with *sfgfp* to stringently repress the promoter activity of P_R with the downstream insertion of a LacI-associated operator, LacO. (**b**) and (**c**) display the on- and off-response performance of T-switch and its derivates, respectively, shown in **a** in different growth phases. Recombinant cells were grown for 12 h after changing temperature from 30□ to 37□. In order to demonstrate the bifunctional control performances of T-switch circuits, fluorescence intensity of high temperature-responded (37□) reporter *sfgfp* and low temperature-responded (30□) reporter *mrfp* from cultures in different growth phases (0, 2, 4, 6, 8, 10 or 12 h growths after inoculation) in a 96 deep-well plate, were plotted in part **b** (sfGFP) and **c** (mRFP). '-' Indicates cell cultures maintained at 30□ as controls. Error bars in **b** and **c**, mean ± s.d. of at least three replicates.

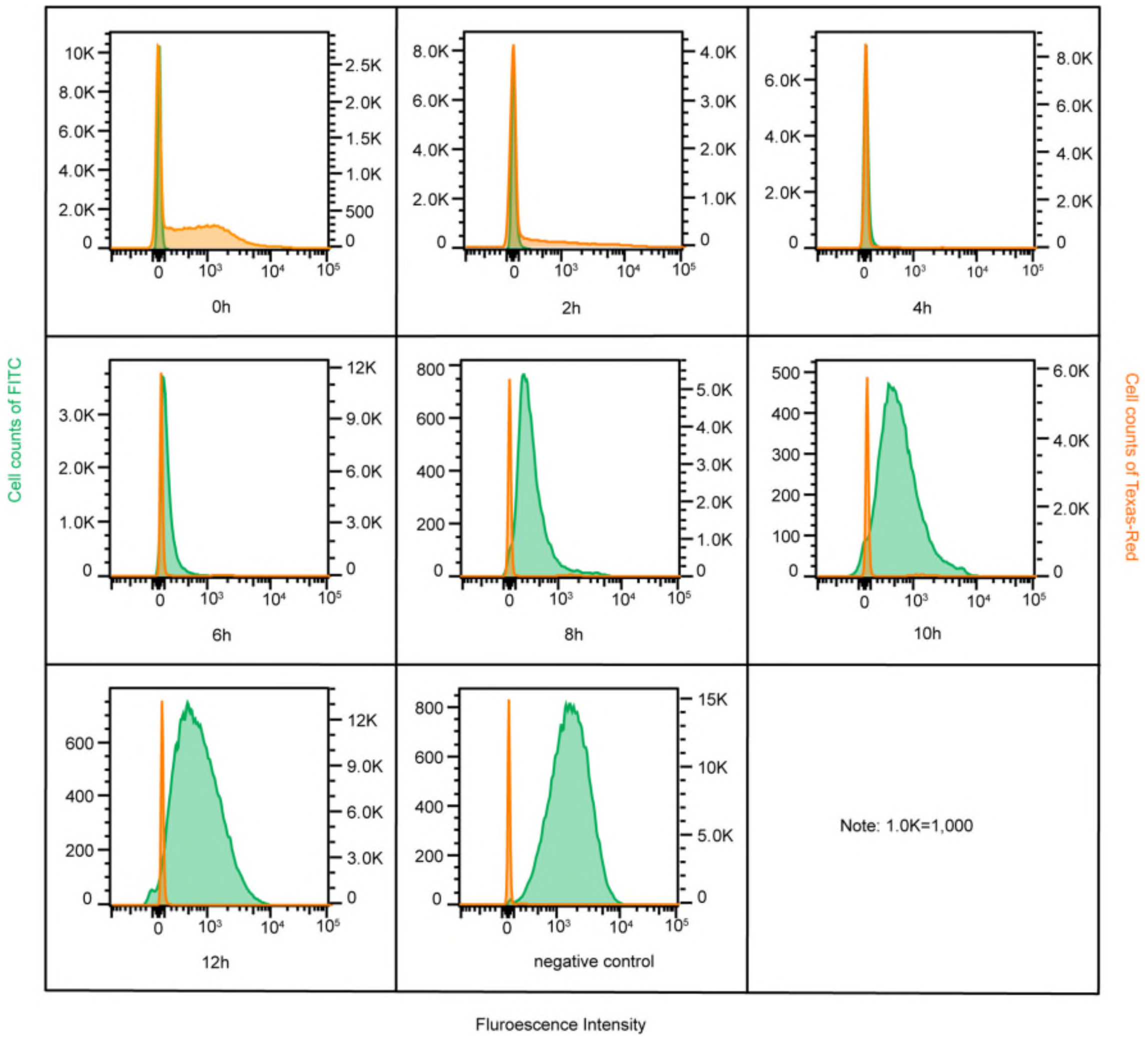

Supplementary Figure 7 Fluorescent distribution of constructs 147+168 in different growth phases

The fluorescent intensity of mRFP of constructs 147+168 group (*mrfp* with AAV tag) shown in Supplementary Figure 6, were plotted against cell counts (density-plot) to illustrate the inconsistency compared to that from other T-switch constructs including the original one, constructs 155+165. Specifically, mRFP fluorescence distribution of the cells captured by Texas-Red channel under thermal induction before 6 h exhibited poor switch-on performance with a lower percentage of positive events. Moreover, on-response cannot be observed under thermal induction after 6 h growth. Negative control group indicates cell cultures maintained at 30℃ before and after inoculation. *y*-axis: cell counts of FITC (left, sfGFP) and Texas-Red (right, mRFP) channels, *x*-axis is logarithmic coordinate of fluorescence intensity of sfGFP and mRFP.

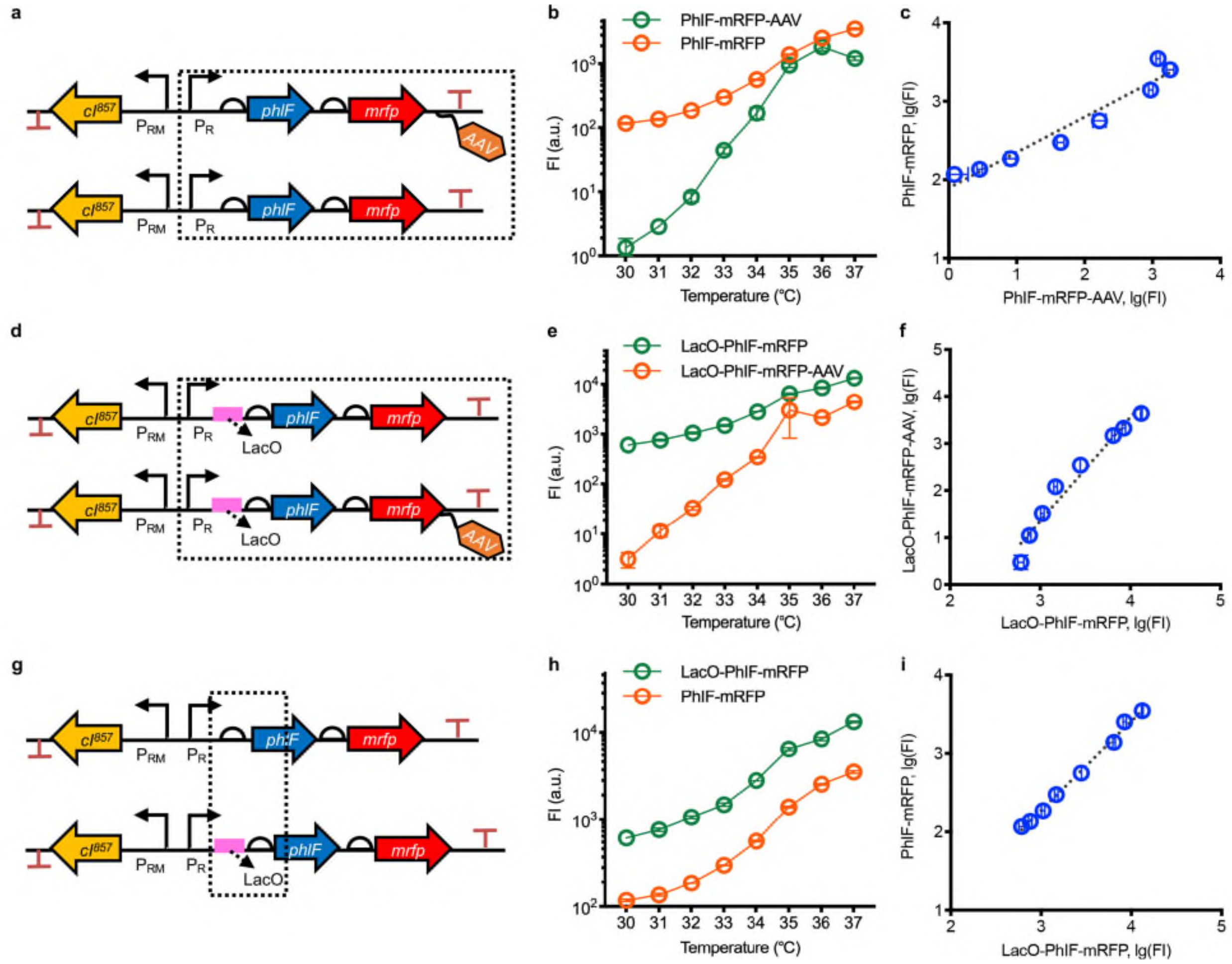

Supplementary Figure 8 Correlations between different designs of the *phlF-mrfp* expression module.

Comparisons of various designs of the *phlF-mrfp* expression module, including the addition of AAV degradation tag to mRFP without (**a**) and with (**d**) LacO insertion downstream of P_R promoter, and the insertion of LacO without the effect of AAV tag (**g**). Fluorescence intensity shown in (**b**), (**e**) and (**h**) were measured from cultures under temperature from 30□ to 37□ for generating temperarure-responsive expression levels of *phlF-mrfp* clusters, and plotted in log-log corrdinate (lg: $\log_{10}$, FI: fluorescence intensity) for linear regression analysis with R^2 = 0.9382 and slope k = 0.4508 in (**c**), R^2 = 0.9580 and slope k = 2.207 in (**f**), and R^2 = 0.9949 and slope k = 1.1390 in (**i**). Notably, the introduction of LacO operator can lead to obvious increase of fluorescence linearly, and the addition of degradation tags will decrease the leakiness at 30□ by approximately 100-folds. Error bars, mean±s.d. of at least three replicates.

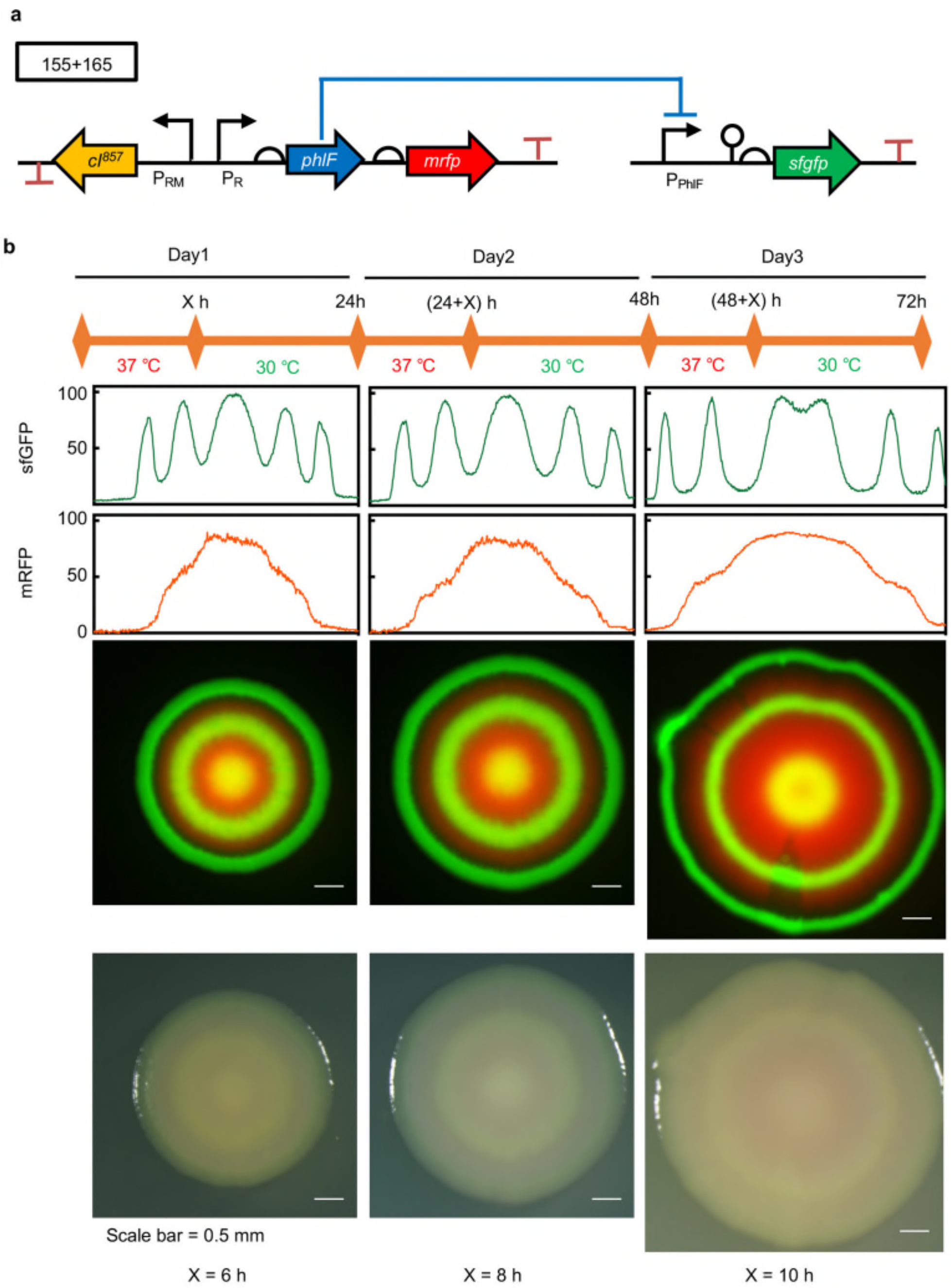

Supplementary Figure 9 Online monitoring of cells containing T-switch constructs from 37□ to 30□.

(**a**) Circuit diagram of T-switch, constructs 155+165. (**b**) The same procedure as shown in Figure 3, while the time cycle maintained at 30□ (*x*) was set at 6, 8 and 10 h, respectively. Accordingly, the cycle time at 37□ was adjusted to (24-*x*) to 18, 16 and 14 h, respectively. Different colony ring patterns were observed with various fluorescence distributions. Fluorescence intensity (FI) was measured by imageJ from images and normalized by the maximum FI value of sfGFP and mRFP measured from every single image, ranging from 0 to 100.

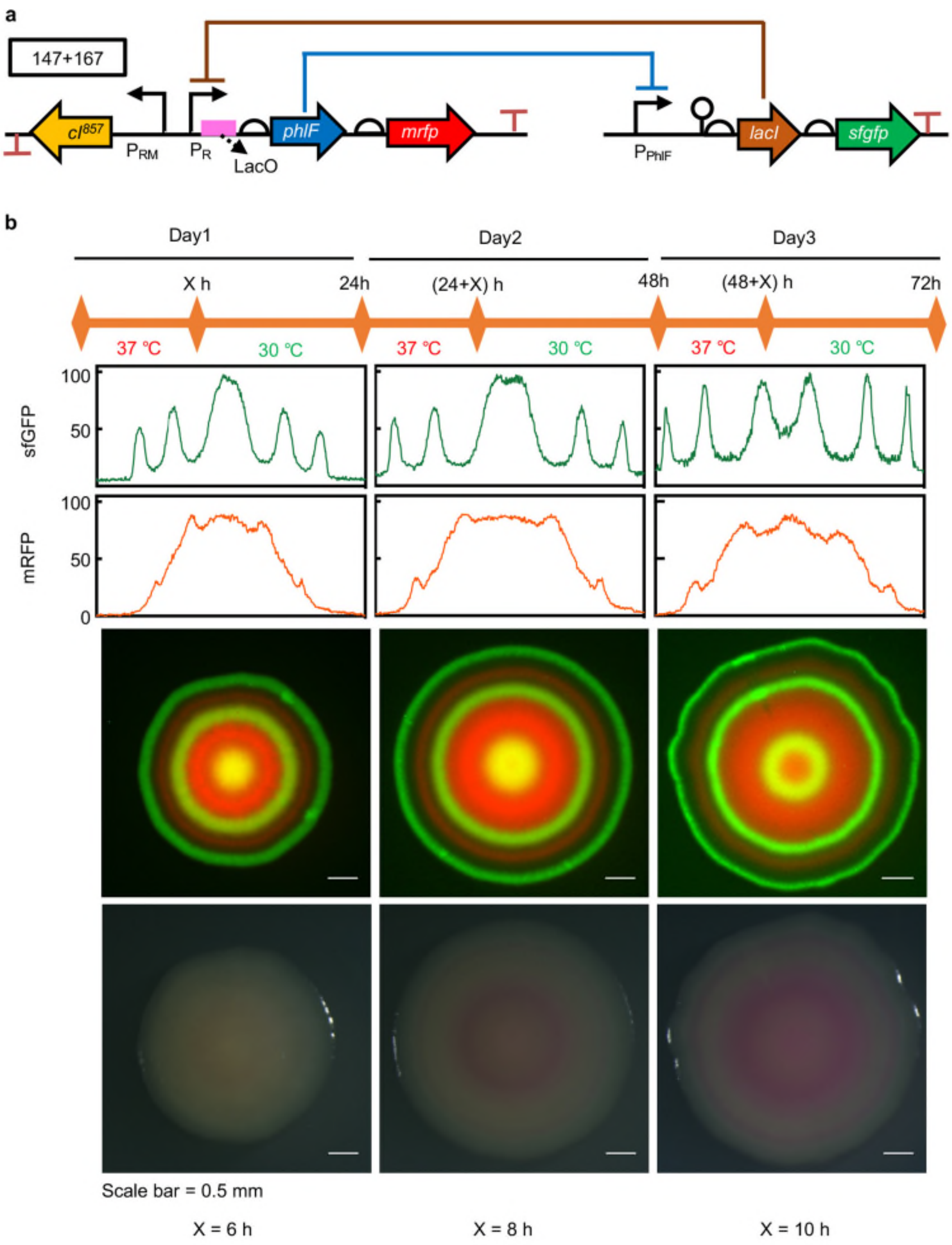

Supplementary Figure 10 Tree-ring like colony formation by *E. coli* harboring T-switch, constructs 147+167.

(**a**) Circuit diagram of T-switch, constructs 147+167. (**b**) The same procedure as shown in Figure 3, while the time cycle maintained at 30□ (*x*) was set at 6, 8 and 10 h, respectively. Accordingly, the cycle time at 37□ was adjusted to (24-*x*) to 18, 16 and 14 h, respectively. Different colony ring patterns were observed with various fluorescence distributions. Fluorescence intensity (FI) was measured by imageJ from images and normalized by the maximum FI value of sfGFP and mRFP measured from every single image, ranging from 0 to 100.

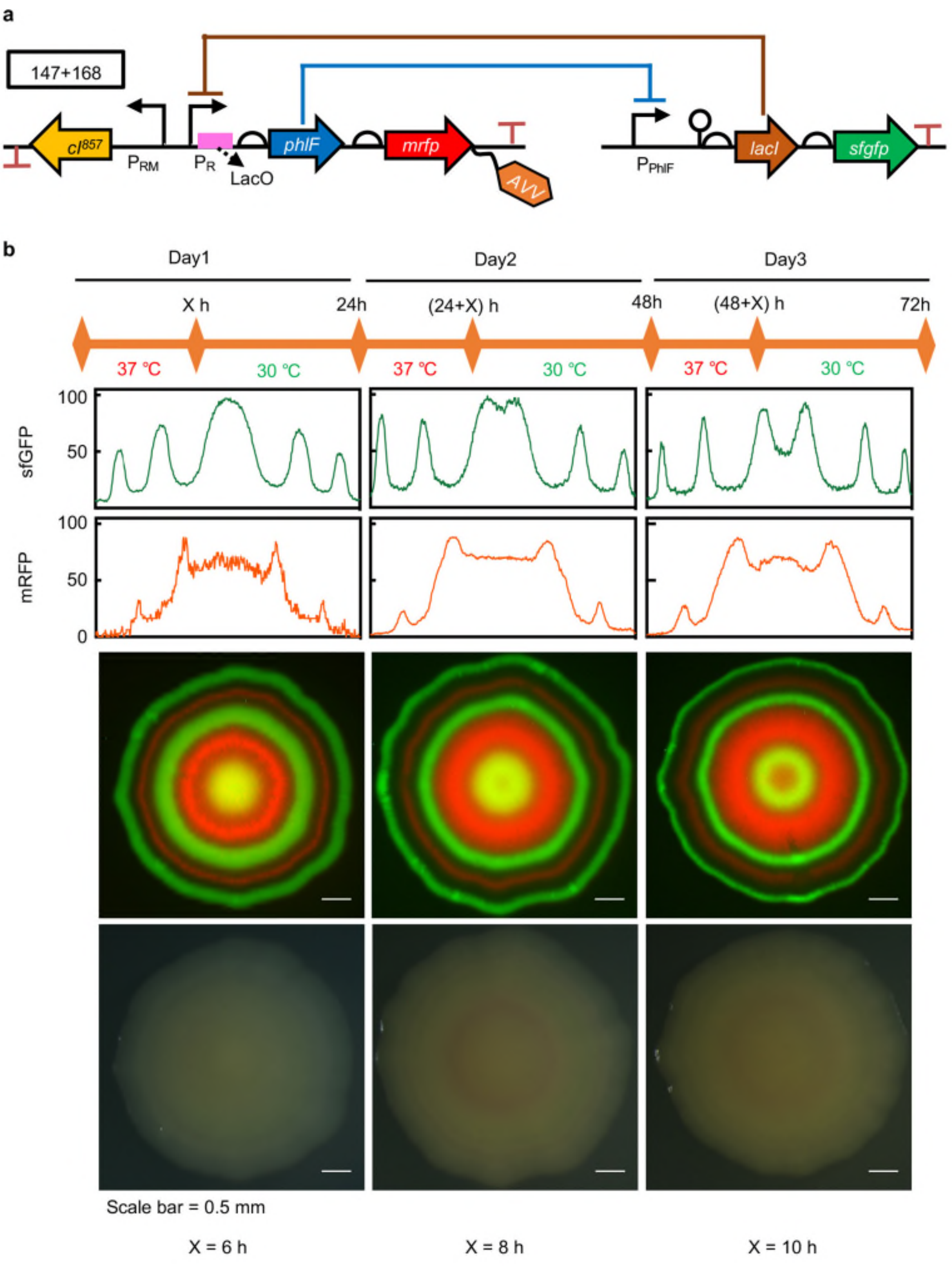

Supplementary Figure 11 Tree-ring like colony formation by *E. coli* harboring T-switch, constructs 147+168.

(**a**) Circuit diagram of T-switch, constructs 147+168. (**b**) The same produre as shown in Supplementary Figure 9. To some extent, colony patterns and fluorescence distributation among the three incubation procedures are slightly different. The boundaries between green and red rings are distinguished with less merged region to form yellow inner-rings (Figure 3 & Supplementary Figure 10). However, fluorescence intensity of mRFP decreased significantly in the outermost ring formation in 3rd day probably due to the joint effects of negative feedback control of *mrfp* expression and fast degradation of mRFP. Fluorescence intensity (FI) was

measured by imageJ from images and normalized by the maximum FI value of sfGFP and mRFP measured from every single image, ranging from 0 to 100.

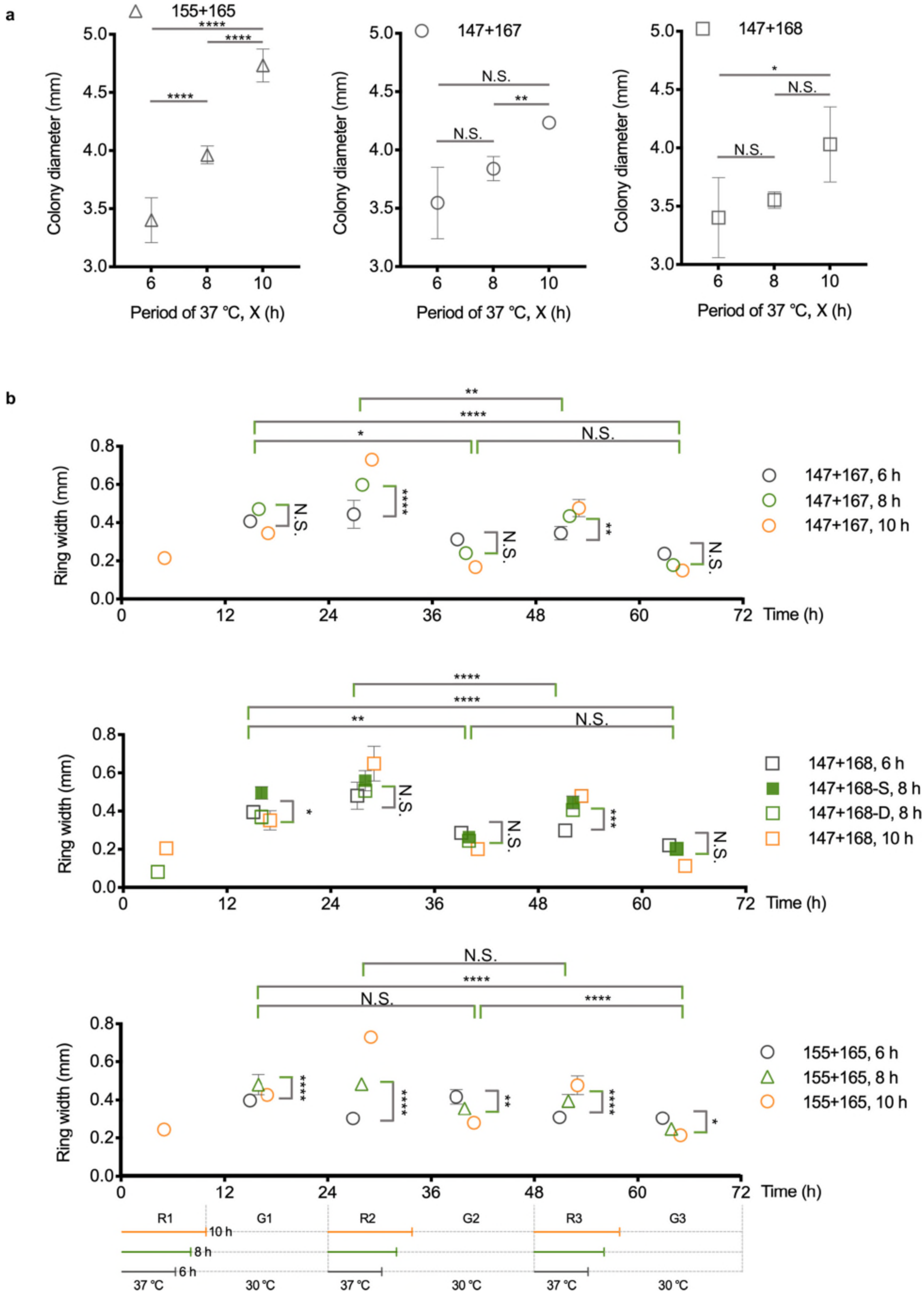
a
155+165
147+167
147+168
Colony diameter (mm)
Period of 37 °C, X (h)
b
Ring width (mm)
Time (h)
147+167, 6 h
147+167, 8 h
147+167, 10 h
147+168, 6 h
147+168-S, 8 h
147+168-D, 8 h
147+168, 10 h
155+165, 6 h
155+165, 8 h
155+165, 10 h
R1
G1
R2
G2
R3
G3
10 h
8 h
6 h
37 °C
30 °C

Supplementary Figure 12 Growth effects on tree ring-like colony formation.

(**a**) Diameter of tree ring-like colonies increased with longer temperature cycles at 37°C (X). (b) Ring width of both sfGFP and mRFP was significantly affected by the colony growth rate (log phase and stationary phase with p value horizontally plotting, X = 8 h) and incubation time X (p value plotted vertically, X = 6 and 8 h). Hollow and solid squares represent the visible and missing formation of the first red ring (R1) generated by constructs 147+167, respectively, namely 147+167-D (2 colonies) and 147+167-S (4 colonies), respectively. One-way ANOVA with Tukey-Kramer test was used in **a** and **b**. p values were partially shown in this figure due to the limited space, all of the one-way ANOVA calculation results are included in Source Data file; N.S. not significant; * $p < 0.0332$; ** $p < 0.0021$; *** $p < 0.0002$; and **** $p < 0.0001$.

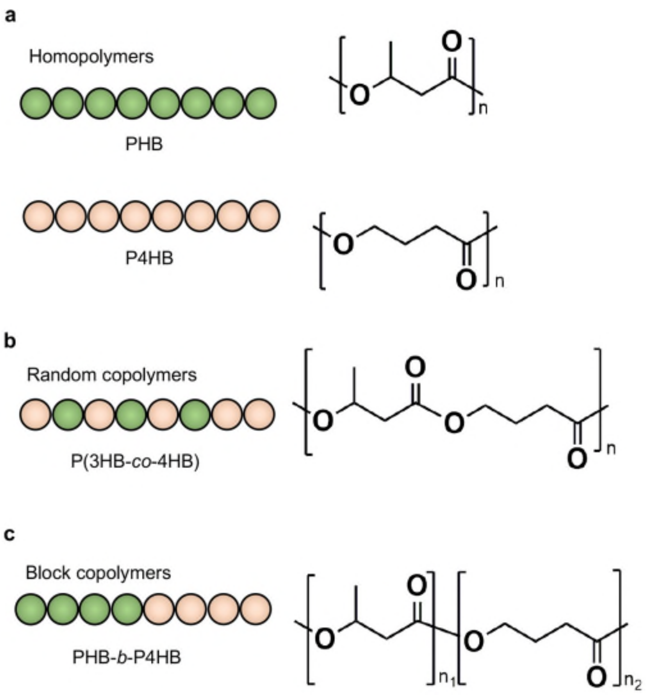

Supplementary Figure 13 Schematics of homopolymer, random- and block-copolymers composed of 3HB and 4HB monomers

(**a**) Homopolymers PHB and P4HB consisting of 3HB (green balls) and 4HB (orange balls) monomer, respectively. (**b**) Random copolymerization of 3HB and 4HB, P(3HB-*co*-4HB), produced by engineered organisms. (**c**) Block copolymerization of 3HB and 4HB units to form block copolymer composed of two homologous modules identical to PHB and P4HB, PHB-*b*-P4HB. The ‘n’ represents the degree of polymerization, which determines the molecular weight of biopolymers.

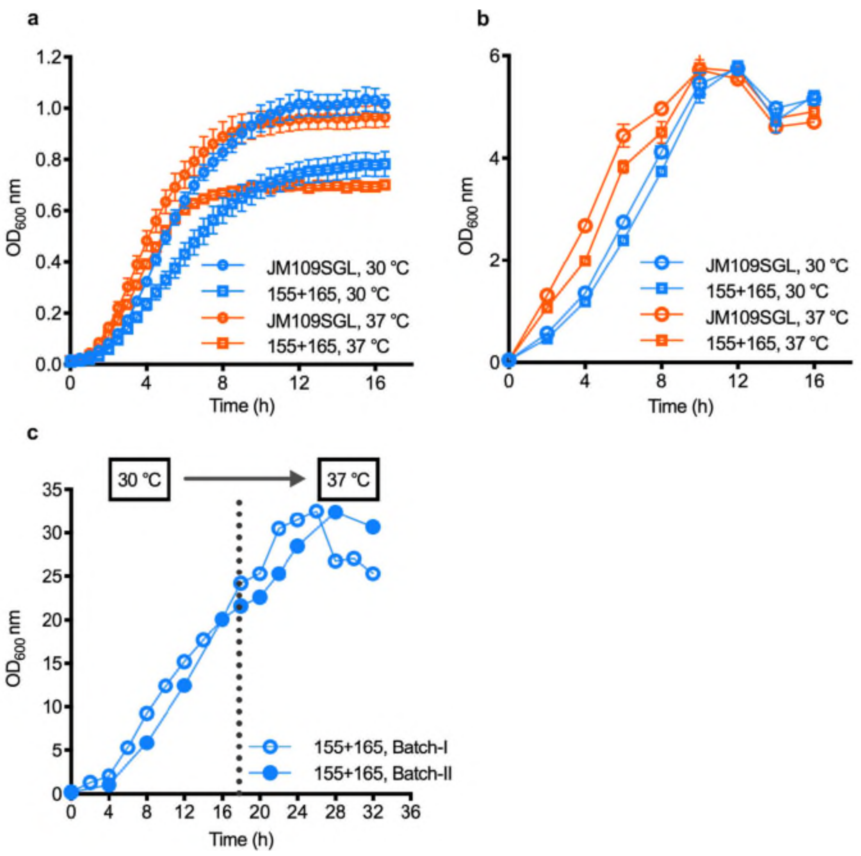

Supplementary Figure 14 Cell growth studies in different culture scales.

(**a**) Growth characterized by online monitoring the OD_{600} form cell cultures, including *E. coli* JM109SGL and its recombinant harboring constructs of 155+165, at 30□ and 37□, respectively, in a 96-well plate containing a LB medium with relevant antibiotics. (**b**) Off-line monitoring of OD_{600} using the same recombinant cells used in **a** grown at 30□ and 37□, respectively, in a 150 ml conical flask containing 20 mL LB medium under the same conditions. (**c**) Studies on cell growth under changing temperatures from 30□ to 37□ at 18 h during fed-batch growths in a 1 L bioreactor using the same cell types as used in **a**. Data in **a** and **b** are presented as mean ± s.d. of three replicates. Two repeated fed-batch fermentations were shown in **c**.

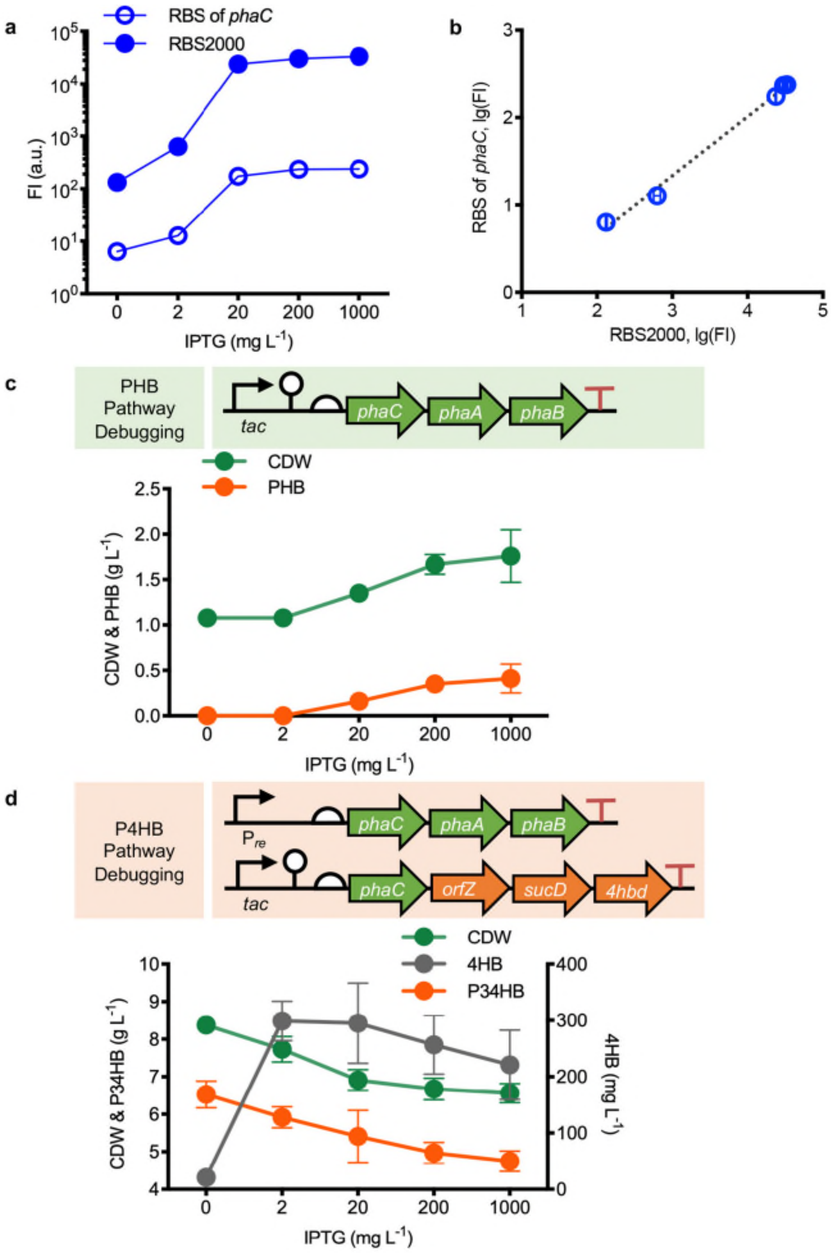

Supplementary Figure 15 Expression debugging of 3HB and 4HB synthsis pathways.

(**a**) Does-responsive functions of *tac* inducible promoter with two different RBS, RBS2000 and RBS from *phaC* gene, which were used for pathway debugging. (**b**) The fluorescence intensity of sfGFP expressed by these two inducible systems were plotted in log-log corrdinate ($\log_{10}$) to determine their linear correlation with an R^2 = 0.9937 and slope k = 0.6776. lg: $\log_{10}$, FI: fluorescence intensity. (**c**) Cell dry weights and PHB produced by recombinant *E. coli* carrying *phaCAB* expression module controlled by *tac* promoter with RBS of *phaC* induced by various concentration of IPTG, 0, 2, 20, 200 and 1000 mg L^{-1}. (**d**) Cell dry weights, P(3HB-*co*-4HB) contents with different 4HB molar fractions produced by *E. coli* JM109SGL harboring *phaCAB* operon under a constitutive *porin* promoter with RBS of *phaC*, and *orfZ-sucD-4hbd* cluster

under the control of *tac* promoter with RBS2000 in the prescence of 0, 2, 20, 200 or 1000 mg L^{-1} IPTG, respectively. Data in **a, c** and **d** are presented as mean ± s.d. of three replicates.

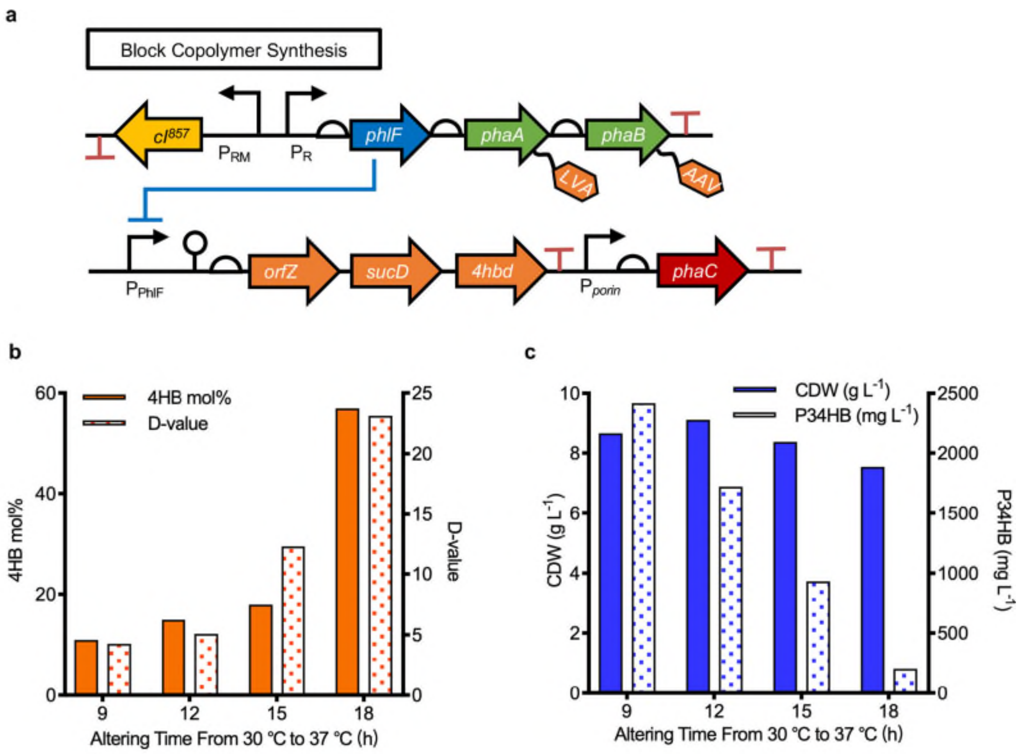

Supplementary Figure 16 Microbial synthesis of block copolymers PHB-*b*-P4HB in a fermentor.

(**a**) Genetic circuit design for production of PHB-*b*-P4HB using P_R promoter with slight leakages (FI of mRFP = 97) to control the expression of *phlF*-*phaA*-*phaB* cluster, which is not sensitive to low expression levels shown in Figure 5 & Supplementaty Figure 15. Accorddingly, *phaA*-*phaB* cluster and *phaC* gene were controlled by a tight regulated promoter P_{phlF} and constitutive *porin* promoter, respectively. (**b**) 4HB molar fractions and D-values of various PHB-*b*-P4HB produced from fed-batch fermetations by changing temperature from 30℃ to 37℃ after 9, 12, 15 and 18 h of growth, respectively, to bidirectionally control the expression of 3HB synthesis pathway and repression of 4HB synthesis pathway dynamically. (**c**) Cell dry weights and production titers of PHB-*b*-P4HB from the experiments shown in part **b**. The time to change temperature from 30℃ to 37℃ could significantly increase the 4HB molar fractions and D-value of PHB-*b*-P4HB, yet exhibiting negative effects on titers and cell dry weights, due to the extended period of 4HB synthsis pathway expression. Fed-batch studies in **b** and **c** were conducted in single batch fermentation of each test group, which means n = 1 in **b** and **c**.

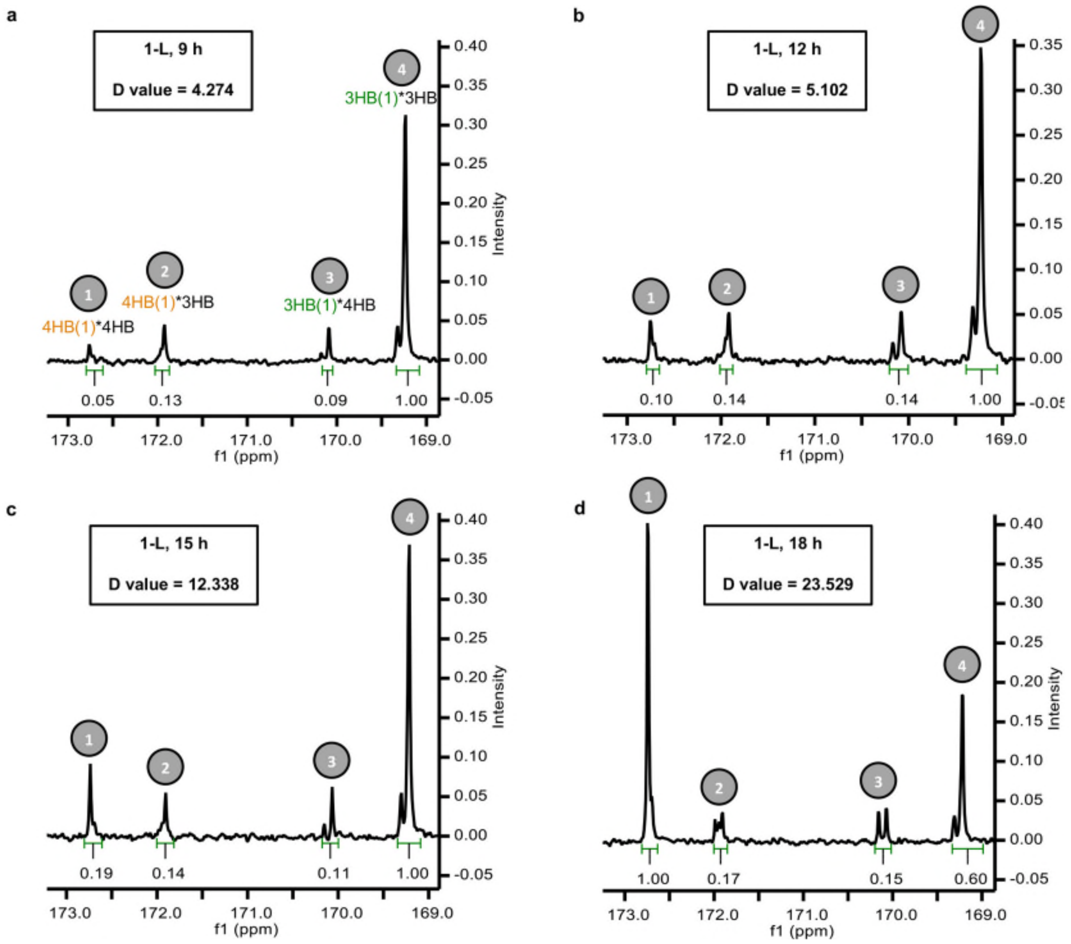

Supplementary Figure 17 NMR study on D-values of PHB-*b*-P4HB produced from fed-batch fermentations.

Characteristic peaks labeled with 1, 2, 3 and 4 highlighted in grape circles indicate four copolymerization combinations of 3HB and 4HB monomers, including 4HB*4HB, 4HB*3HB, 3HB*4HB, 4HB*4HB, respectively. PHB-*b*-P4HB productions from fed-batch fermetations by changing temperature from 30□ to 37□ at 9 h (**a**), 12 h (**b**), 15 h (**c**) and 18 h (**d**), respectively, during the cell growths, exhibited increased D-values from 4.274 to 23.529. Generally, copolymer with D-value over 10 are regarded as block copolymer.

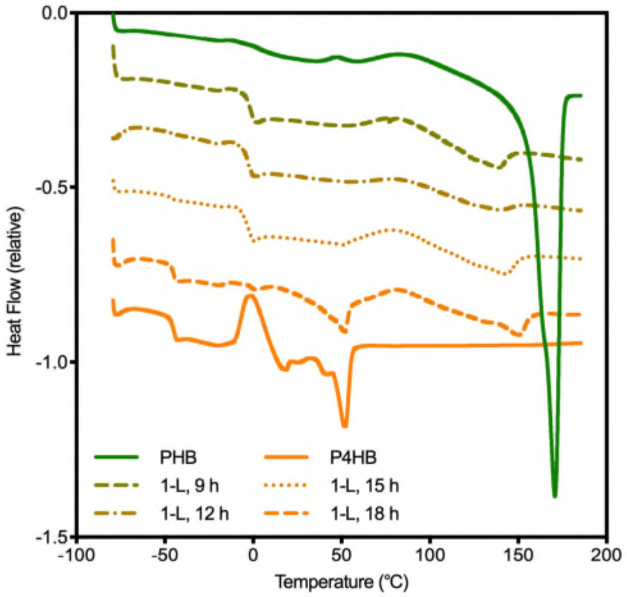

Supplementary Figure 18 DSC thermodynamic characterization of PHB-*b*-P4HB.

DSC was carried out using PHB-*b*-4HB generated from experiments shown in Supplementary Figures 16 & 17, together with homopolymers PHB (solid line in green) and P4HB (solid line in orange), as controls. The PHB-*b*-P4HB with higher D-values display more arresting peaks of two melting points that are identical to PHB and P4HB homopolymers.

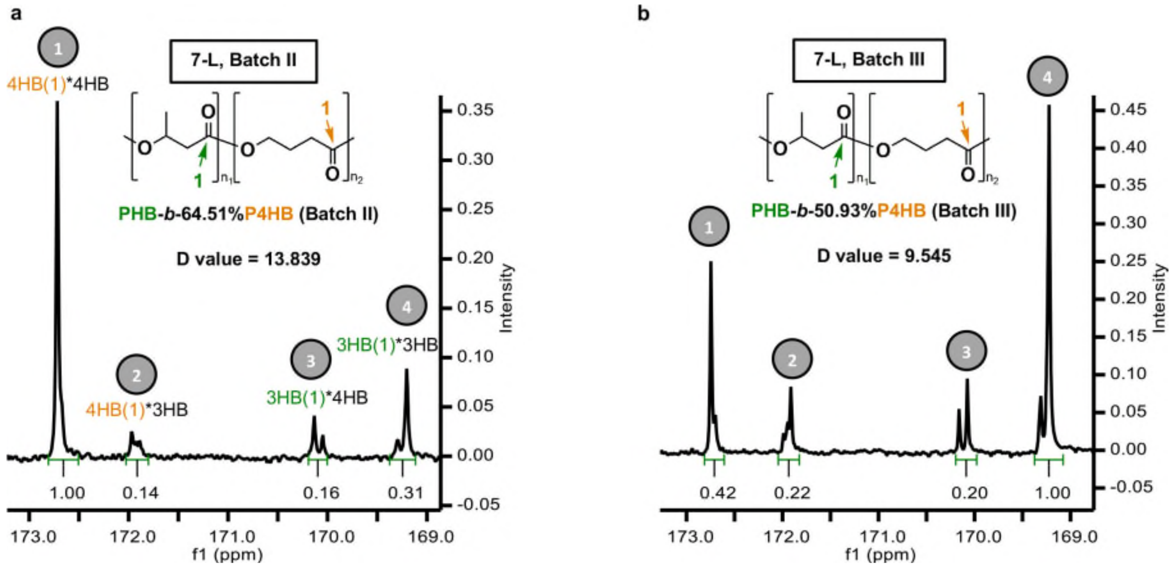

Supplementary Figure 19 NMR study on D-values of PHB-*b*-P4HB produced from fed-batch fermentations.

NMR results of PHB-*b*-P4HB obtained from Batch-II (**a**) and Batch-III (**b**) fermentations (Figure 5) for D-value calculation. D-values of PHB-*b*-P4HB containing 65 mol% and 50.1 mol% 4HB from Batch-II and Batch-III, respectively, are 13.839 and 9.545, respectively, close to 10 considered as block copolymers.

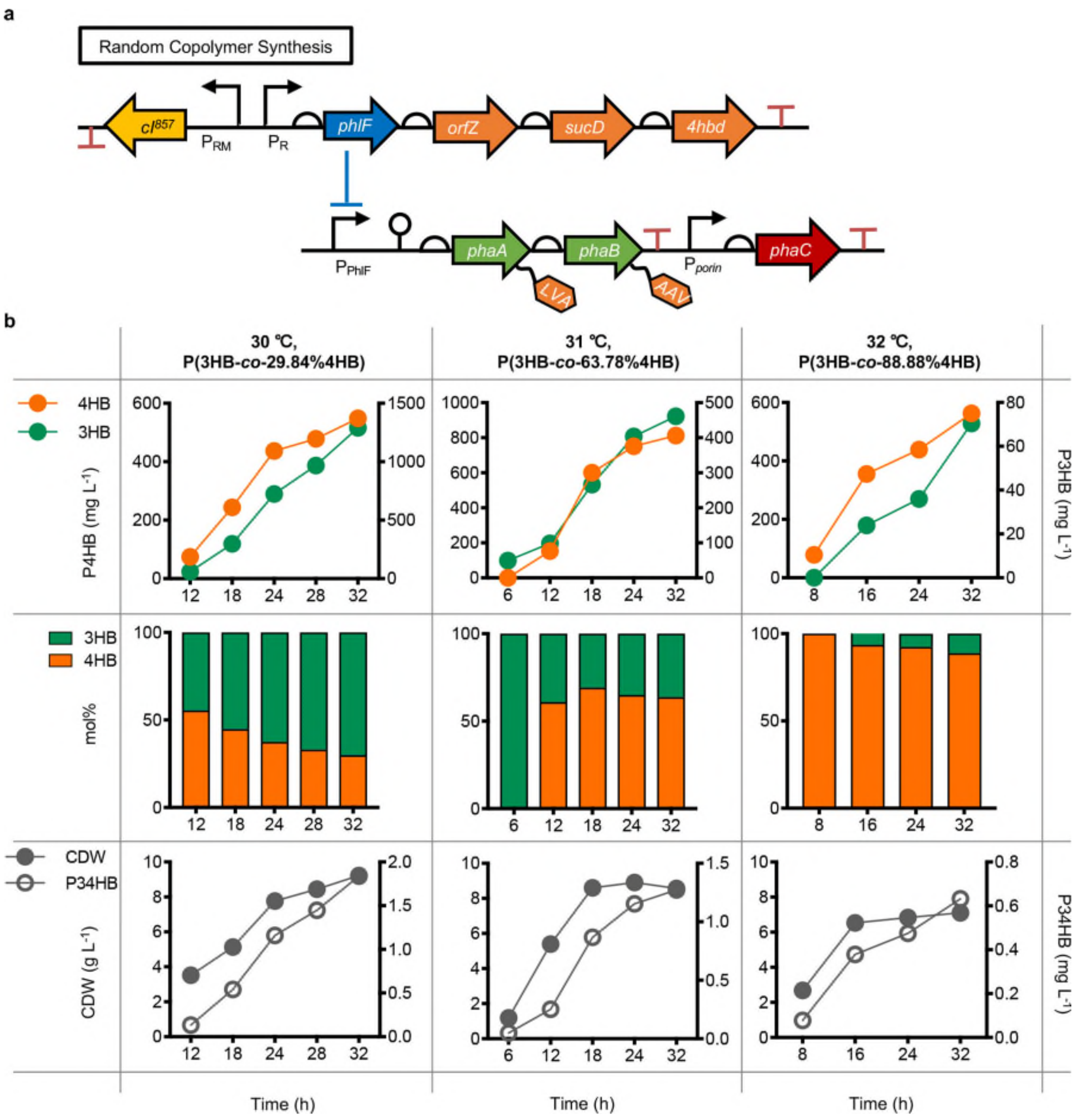

Supplementary Figure 20 Microbial synthesis of random copolymer P(3HB-*co*-4HB) by recombinant *E. coli* grown in a fermentor.

(**a**) Genetic circuit for production of P(3HB-*co*-4HB) using P_R promoter with leakages (FI of mRFP = 97) to control the expression of *orfZ-sucD-4hbd* cluster, which is sensitive to low expression levels shown in Supplementary Figure 15. Accorddingly, *phaA-phaB* cluster and *phaC* gene were controlled by a tight regulated promoter P_{PhlF} and constitutive *porin* promoter, respectively. (**b**) Random copolymers of P(3HB-*co*-4HB) from three individual batches of fermentation at different temperatures for various combinations of expression and repression activities of *orfZ-sucD-4hbd* and *phaA-phaB* gene clusters, respectively. Accordingly, P(3HB-*co*-4HB) containing 30 mol%, 64 mol% and 89 mol% 4HB were produced by recombinant *E. coli* cultured at 30□, 31□ and 32□, respectively, via statically comtrolling the expression levels of *orfZ-sucD-4hbd* and *phaA-phaB* gene clusters. Upper panel displays the

accumulations of 3HB and 4HB in P(3HB-*co*-4HB) during the bioprocesses; middle panel shows 4HB molar fractions of time course; bottom panel cell dry weight and PHA content profiling throughout the growths. All of the data exihibit remarkable differences compared to that from block copolymers synthesis (Figure 5).

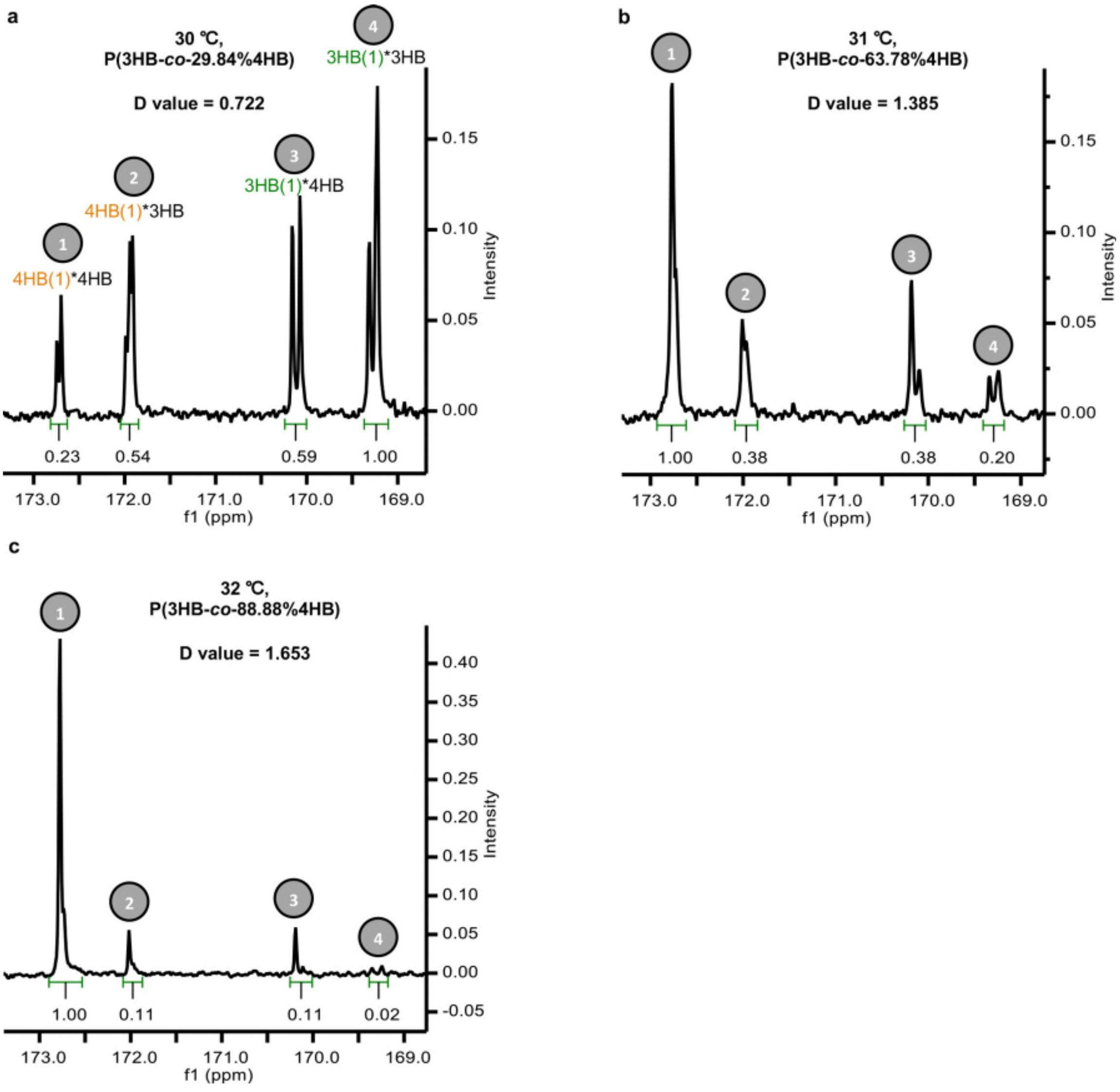

Supplementary Figure 21 NMR study on D-values of P(3HB-*co*-4HB) from fed-batch fermentations.

Random copolymer P(3HB-*co*-4HB) produced from fed-batch cultivations at 30□ (**a**), 31□ (**b**), and 32□ (**c**), respectively, throughout the growth. The resulted D-values of the copolymers are closed to 1, at 0.722 in **a**, 1.385 in **b**, and 1.653 in **c**, suggesting that all the PHA as random copolymers.

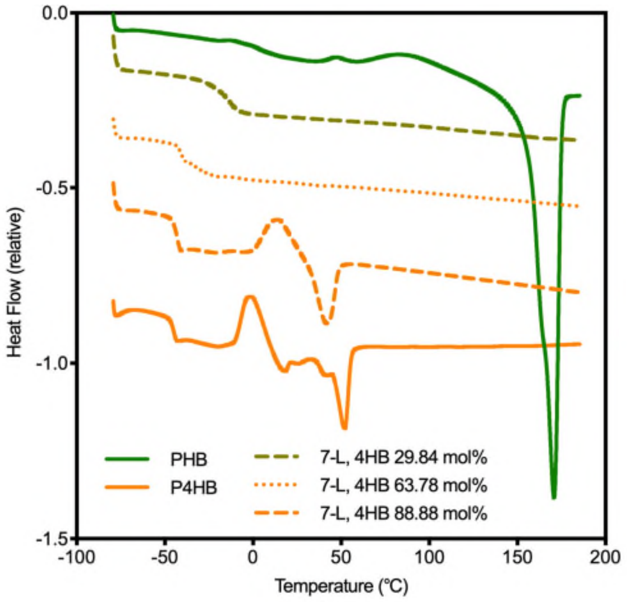

Supplementary Figure 22 DSC thermodynamic assays of P(3HB-*co*-4HB).

DSC were performed using P(3HB-*co*-4HB) from samples obtained in Supplementary Figure 20. Homopolymers including PHB (solid line in green) and P4HB (solid line in orange) were used as controls. P(3HB-*co*-4HB) consisting of 30 mol% and 64 mol% 4HB monomer fraction did not exhibit arresting melting points (T_m), however, the PHA containing 89 mol% 4HB ratio displays similar T_m value compared to P4HB homopolymer for its extremely high molar fraction of 4HB.

Supplementary References

1. Wu, J. J., Buckley, C. P. & O'Connor, J. J. Mechanical integrity of compression-moulded ultra-high molecular weight polyethylene: Effects of varying process conditions. *Biomaterials* **23**, 3773–3783 (2002).
2. Li, Z. J. *et al.* Production of poly(3-hydroxybutyrate-*co*-4-hydroxybutyrate) from unrelated carbon sources by metabolically engineered *Escherichia coli*. *Metab. Eng.* **12**, 352–359 (2010).
3. Ye, J. *et al.* Engineering of *Halomonas bluephagenesis* for low cost production of poly(3-hydroxybutyrate-*co*-4-hydroxybutyrate) from glucose. *Metab. Eng.* **47**, 143–152 (2018).
4. Jiang, X. R., Yao, Z. H. & Chen, G.-Q. Controlling cell volume for efficient PHB production by *Halomonas*. *Metab. Eng.* **44**, 30–37 (2017).
5. Ye, J. *et al.* Stimulus response-based fine-tuning of polyhydroxyalkanoate pathway in *Halomonas*. *Metab. Eng.* **57**, 85–95 (2020).
6. Elvin, C. M. *et al.* Modified bacteriophage lambda promoter vectors for overproduction of proteins in *Escherichia coli*. *Gene* **87**, 123–126 (1990).
7. Stanton, B. C. *et al.* Genomic mining of prokaryotic repressors for orthogonal logic gates. *Nat. Chem. Biol.* **10**, 99–105 (2014).
8. Campbell, R. E. *et al.* A monomeric red fluorescent protein. *Proc. Natl. Acad. Sci. U. S. A.* **99**, 7877–7882 (2002).
9. Zhao, H. *et al.* Novel T7-like expression systems used for *Halomonas*. *Metab. Eng.* **39**, 128–140 (2017).
10. Karzai, A. W., Roche, E. D. & Sauer, R. T. The SsrA-SmpB system for protein tagging, directed degradation and ribosome rescue. *Nat. Struct. Biol.* **7**, 449–455 (2000).
