# Supplementary material for: Reversible thermal regulation for bifunctional dynamic control of gene expression in Escherichia coli
Source: Nat Commun. 2021 Mar 3;12:1411. doi: 10.1038/s41467-021-21654-x (PMC7930084; doi:10.1038/s41467-021-21654-x)
Supplement: Supplementary file 12 — Description of Additional Supplementary Files [file 41467_2021_21654_MOESM12_ESM.pdf]

Supplementary Data 1:
Plasmids and primers used in this study
Supplementary Data 2:
OD_{600} of Fed-batch Fermentation

Supplementary Movie 1: Cell growth of recombinant *E. coli* JM109SGL harboring 155+165 T-switch circuit
E. coli JM109SGL harboring 155+165 constructs (described in Fig. 2a) were cultured from single colony at 37°C overnight and then transferred under the agar tablet for continuously monitoring using confocal microscopy at 30°C for 12 hours. The expression of mRFP (color in red) was inhibited and completely disappeared in 6-8 hours, followed by the expression of sfGFP (color in green). Images were captured every 10 minutes. Scale bar = 2μm.

Supplementary Movie 2: Explanation of the formation of tree ring-like colonies
Explain the different patterns of colony rings in green and/or red color during the three-day formation of tree ring-like colony (Fig. 3d). The colony growth under periodic changes of temperature at 30 and 37°C along with the expression of sfGFP (left), mRFP (middle) and the merged effect (right) were shown together in the video.

Supplementary Movie 3: Images used for quantitative measurements of cell lengths of Fig. 3c (30°C)
E. coli JM109SGL harboring constructs 145+221 (Fig. 3a) exhibited spherical shapes when cultured at 30°C. Original images collected to gather the cell length distribution (Fig. 3c). The video contains 10 random selected images containing at least 150 cells for cell length measurements. Scale bar = 2μm.

Supplementary Movie 4: Images used for quantitative measurements of cell lengths of Fig. 3c (33°C)
E. coli JM109SGL harboring constructs 145+221 (Fig. 3a) exhibited normal rods shapes when cultured at 33°C. Original images collected to gather the cell length distribution (Fig. 3c). The video contains 10 random selected images containing at least 150 cells for cell length measurements. Scale bar = 2μm.

Supplementary Movie 5: Images used for quantitative measurements of cell lengths of Fig. 3c (35°C)
E. coli JM109SGL harboring constructs 145+221 (Fig. 3a) exhibited fiber shapes when cultured at 35°C. Original images collected to gather the cell length distribution (Fig.

3c). The video contains 10 random selected images containing at least 150 cells for cell length measurements. Scale bar = 2μm.

Supplementary Movie 6: Dynamic control of cell shapes of Fig. 3d (30°C overnight for seed preparation, then 35°C for 8 h)
E. coli JM109SGL harboring constructs 145+221 (described in Fig. 3a) were pre-cultured overnight from single colony, and then transferred into microfluidic device for single cell cultivation and monitoring: 30°C overnight for seed preparation, then 35°C for 8 h. Images were captured in every 3 minutes. Scale bar = 2μm.

Supplementary Movie 7: Dynamic control of cell shapes of Fig. 3d (33°C overnight for seed preparation, then 35°C for 8 h)
E. coli JM109SGL harboring constructs 145+221 (described in Fig. 3a) were pre-cultured overnight from single colony, and then transferred into microfluidic device for single cell cultivation and monitoring: 33°C overnight for seed preparation, then 35°C for 8 h. Images were captured in every 3 minutes. Scale bar = 2μm.

Supplementary Movie 8: Dynamic control of cell shapes of Fig. 3d (30°C 1.5 h, followed by 33°C 2.5 h, then 35°C 4 h)
E. coli JM109SGL harboring constructs 145+221 (described in Fig. 3a) were pre-cultured overnight from single colony, and then transferred into microfluidic device for single cell cultivation and monitoring: 30°C overnight for seed preparation, 30°C for 1.5 h, followed by 33°C for 2.5 h, then 35°C for the remaining 4 h (8 h cultivation in total). Images were captured in every 3 minutes. Scale bar = 2μm.
