# Supplementary material for: Reversible thermal regulation for bifunctional dynamic control of gene expression in Escherichia coli
Source: Nat Commun. 2021 Mar 3;12:1411. doi: 10.1038/s41467-021-21654-x (PMC7930084; doi:10.1038/s41467-021-21654-x)
Supplement: Supplementary file 13 — Reporting Summary [file 41467_2021_21654_MOESM13_ESM.pdf]

nature research

Corresponding author(s): Jian-Wen Ye; Guo-Qiang Chen

Last updated by author(s): Jan 28, 2021

Reporting Summary

Nature Research wishes to improve the reproducibility of the work that we publish. This form provides structure for consistency and transparency in reporting. For further information on Nature Research policies, see our Editorial Policies and the Editorial Policy Checklist.

Statistics

For all statistical analyses, confirm that the following items are present in the figure legend, table legend, main text, or Methods section.

n/a	Confirmed	
[ ]	[x]	The exact sample size (*n*) for each experimental group/condition, given as a discrete number and unit of measurement
[ ]	[x]	A statement on whether measurements were taken from distinct samples or whether the same sample was measured repeatedly
[x]	[ ]	The statistical test(s) used AND whether they are one- or two-sided<br>*Only common tests should be described solely by name; describe more complex techniques in the Methods section.*
[x]	[ ]	A description of all covariates tested
[x]	[ ]	A description of any assumptions or corrections, such as tests of normality and adjustment for multiple comparisons
[ ]	[x]	A full description of the statistical parameters including central tendency (e.g. means) or other basic estimates (e.g. regression coefficient) AND variation (e.g. standard deviation) or associated estimates of uncertainty (e.g. confidence intervals)
[x]	[ ]	For null hypothesis testing, the test statistic (e.g. *F*, *t*, *r*) with confidence intervals, effect sizes, degrees of freedom and *P* value noted<br>*Give P values as exact values whenever suitable.*
[x]	[ ]	For Bayesian analysis, information on the choice of priors and Markov chain Monte Carlo settings
[x]	[ ]	For hierarchical and complex designs, identification of the appropriate level for tests and full reporting of outcomes
[ ]	[x]	Estimates of effect sizes (e.g. Cohen's *d*, Pearson's *r*), indicating how they were calculated

Our web collection on statistics for biologists contains articles on many of the points above.

Software and code

Policy information about availability of computer code

Data collection	The cell growth data were collected using a micro-plate reader (Varioskan Flash, Thermo Scientific), and the other data were collected by commercial equipments and softwares shown in Methods and Materials section.
Data analysis	Genetic design and sequence reading were performed by SnapGene v3.2.1. Flow cytometer data were analyzed by FlowJo v10.7 software.µManager v1.446 and NIS elements v4.60 were use to process video of mophology change and fluorescence change. MestReNova12 was used for spectra analysis. Figures were generated through Adobe Illustrator CC2017, Prism v8 (Graphpad), Microsoft Office 2016 (Power Point, Excel and Word) and ImageJ whatever necessary.

For manuscripts utilizing custom algorithms or software that are central to the research but not yet described in published literature, software must be made available to editors and reviewers. We strongly encourage code deposition in a community repository (e.g. GitHub). See the Nature Research guidelines for submitting code & software for further information.

Data

Policy information about availability of data

All manuscripts must include a data availability statement. This statement should provide the following information, where applicable:

- Accession codes, unique identifiers, or web links for publicly available datasets
- A list of figures that have associated raw data
- A description of any restrictions on data availability

The authors declare that source data processed for figure generation in this study are available within the paper and its Supplementary Information files. The source data underlying Figures 1c-e, 2b-d, 3b-e, 4b-d, 5b-e, as well as Supplementary Table 3 and Supplementary Figures 1, 2, 3c-d, 4a-c, 5, 6b-d, 7, 8b-c, 8e-f, 8h-i, 9b, 10b, 11b, 12, 13, 15, 16b-c, 17a-d, 18, 19, 20b, 21 and 22 are provided as a Source Data file. Plasmids used in this study, OD600 measured in fermentation experiments are deposited in Source Data file. The datasets generated and analyzed during the current study are available from the corresponding authors upon request. A

reporting summary for this article is available as a Supplementary Information file.

Field-specific reporting

Please select the one below that is the best fit for your research. If you are not sure, read the appropriate sections before making your selection.

- [x] Life sciences
- [ ] Behavioural & social sciences
- [ ] Ecological, evolutionary & environmental sciences

For a reference copy of the document with all sections, see nature.com/documents/nr-reporting-summary-flat.pdf

Life sciences study design

All studies must disclose on these points even when the disclosure is negative.

Sample size	The sample sizes of tree ring-like colony assays on agar plate were 2 to 10 due to the dilution (Fig. 3d-e). We used a microfluidic chip to control cell morphology by changing temperature and the sample sizes differs from 4 to 580 due to initial cell numbers and the capacity of the chip (Fig. 4c-d). Fed-batch studies in 1-L fermenters were carried out in single batch, while 3 repeat batches fermentation in 7-L bio-reactors (data was display separately in main text).
Data exclusions	No data exclusions.
Replication	3 biological independent replicates experiments: Fluorescence intensity measurement by flow cytometer, shake flask experiments, growth curve(2 repeat batches in 1-L bioreactor) and mechanical properties of materials. Experimental data were presented as Mean ± S.D. value (clear distribution of data points are shown in histogram plots). Fed-batch studies in 1-L fermenters were one batch each while in 7-L fermenters were three batches (data was display separately in main text). DSC and NMR were measured once each group.
Randomization	No randomization was performed in the study, since all replicates are processed to be test or measured in this study.
Blinding	Blinding was not performed in the study. Because the experiments did not involve any animals or human participants. For cell culturing, seed cells are cultured from single colonies and then inoculated into different 1 mL 96-well plate or 20 mL shake flask or 7-L bioreactor described in the manuscript. And the results are objective.

Reporting for specific materials, systems and methods

We require information from authors about some types of materials, experimental systems and methods used in many studies. Here, indicate whether each material, system or method listed is relevant to your study. If you are not sure if a list item applies to your research, read the appropriate section before selecting a response.

Materials & experimental systems

n/a	Involved in the study	
x		Antibodies
x		Eukaryotic cell lines
x		Palaeontology and archaeology
x		Animals and other organisms
x		Human research participants
x		Clinical data
x		Dual use research of concern

Methods

n/a	Involved in the study	
x		ChIP-seq
	x	Flow cytometry
x		MRI-based neuroimaging

Flow Cytometry

Plots

Confirm that:

- [x] The axis labels state the marker and fluorochrome used (e.g. CD4-FITC).
- [x] The axis scales are clearly visible. Include numbers along axes only for bottom left plot of group (a 'group' is an analysis of identical markers).
- [x] All plots are contour plots with outliers or pseudocolor plots.
- [x] A numerical value for number of cells or percentage (with statistics) is provided.

Methodology

Sample preparation	To characterize the performance of various designs of T-switch (Supplementary File 9), all measurements of fluorescence intensity were taken by cytometer of cells in the beginning of stationary phase cultured in 96 deep-well plates. Glycerol stocks of the start host, E. coli JM109SGL, containing target plasmids were streaked and activated on LB agar plates for 12 h

incubation at 37℃. Single colonies were inoculated into 1 mL LB medium, followed by 12 h pre-culture in 2 mL well in 96 deep-well plates (NEST, China) sealed with an air permeable film (Axygen, USA) at 1,000 rpm at different temperatures (30, 31, 32, 33, 34, 35, 36, and 37 ℃), respectively (Thermal Shaker, AOSHENG, China). Then, pre-cultures were 200-folds diluted into 1 mL fresh LB medium for 12 h growth under the same conditions. After growth, cell cultures were 100-folds diluted into 250 μL PBS supplemented with 2 mg mL^-1 kanamycin to terminate the expression of proteins. 0.1 vol% of 50 mg mL^-1 Kan and 25 mg mL^-1 Cm stock solution were added into the cultures for stabilizing plasmids of interest throughout the cultivation processes.

To study the time course responses of T-switch designs, including the combinations of constructs 155+165, 147+167 and 163 +166, recombinant E. coli JM109SGL harboring plasmids of interest were grown overnight on LB agar plates at 30℃. After growth, single colonies were inoculated into 1 mL LB medium in 96 deep-well plates sealed with an air permeable film and grown for 12 h at 1,000 rpm at 30℃ in a Thermal Shaker. Then, 5 μL of each culture was inoculated into 1 mL fresh LB medium for 12 h cultivation under the same conditions. Followed by 200-folds dilution in 1 mL fresh LB medium, cells were grown for 12 h at 1,000 rpm at 37℃. During the growth, 2-10 μL of each culture was sampled in every 1 h and mixed with 250 μL of PBS supplemented with 2 mg mL^-1 Kan. 0.1 vol% of Kan and Cm stock solution were added into the medium for stabilizing plasmids of interest throughout the cultivation processes. Fluorescence intensities of sfGFP and mRFP were measured by FACS.

The on/off performances of T-switch circuits in different growth phases were characterized by cytometer analysis. Single colonies of E. coli JM109SGL harboring plasmids of interest were grown on LB agar plates from glycerol stocks, and then inoculated into 1 mL LB medium for 12 h cultivation (1,000 rpm, 30℃, Thermal Shaker). After growth, 5 μL of each culture was transferred into 1 mL fresh LB media as inoculums. After 12 h growth under the same conditions, 5 μL cultures were diluted into 1 mL fresh LB medium and grown at 30℃ at 1,000 rpm for 0, 2, 4, 6, 8, 10 and 12 h, respectively, then cultures were transferred to new shaker at 37℃ at 1,000 rpm for a 12 h cultivation. Finally, cell cultures were 100-folds diluted into 250 μL PBS supplemented with 2 mg mL^-1 Kan. 0.1 vol% of Kan, and Cm stock solution were added into the medium for stabilizing plasmids of interest throughout the cultural processes. Fluorescence intensities of sfGFP and mRFP were measured by FACS.

Instrument

Flow Cytometer (LSRFortessa4, BD bioscience, USA)

Software

FlowJo (v10.7) software was used to precess the raw data for obtaining mean value of fluorescence intensity and percentage of fluorescent positive cells.

Cell population abundance

2-10 μL culture was added into 250 μL PBS supplemented with 2 mg mL^-1 Kan. The diluted culture was recorded by flow cytometer at the rate of 0.5 μL s^-1 for 20 seconds at least 10,000 cell counts were captured.

Gating strategy

Fluorescence positive cells were captured under the excitation spectrum of 488 nm (FITC channel, 440 V, sfGFP) and 584 nm (PE-Texas Red channel, 580 V, mRFP), the channels of FSC (forward scatter, 440V) and SSC (side scatter, 260V). Furthermore, cells were firstly gated by forward scatter (FSC) and side scatter (SSC) channels (varied by different temperature) to illuminate noise events. Subsequently, fluorescence positive events were determined by fluorescence channels of FITC and Texas-red, respectively, to remove the fluorescence negative cells. Over 90% of cell counts (over 10,000 cell counts) were firstly gated by SSC and FSC channels, of which the fluorescence positive cells were selected by FITC channel for calculating the mean value of fluorescence intensity of GFP and RFP. (Detailed information of the gating strategy displayed in Source Data.)

☒ Tick this box to confirm that a figure exemplifying the gating strategy is provided in the Supplementary Information.
